# Mitochondrial genome annotation and phylogenetic placement of *Oreochromis andersonii* and *O*. *macrochir* among the cichlids of southern Africa

**DOI:** 10.1371/journal.pone.0203095

**Published:** 2018-11-27

**Authors:** Ian Bbole, Jin-Liang Zhao, Shou-Jie Tang, Cyprian Katongo

**Affiliations:** 1 Department of Fisheries, Mansa, Zambia; 2 Key Laboratory of Freshwater Aquatic Genetic Resources, Ministry of Agriculture, Shanghai Ocean University, Shanghai, China; 3 Centre for Research on Environmental Ecology and Fish Nutrition (CREEFN), Ministry of Agriculture, Shanghai Ocean University, Shanghai, China; 4 National Demonstration Centre for Experimental Fisheries Science Education, Shanghai Ocean University, Shanghai, China; 5 Biological Sciences Department, University of Zambia, Lusaka, Zambia; Natural History Museum of London, UNITED KINGDOM

## Abstract

Genetic characterization of southern African cichlids has not received much attention. Here, we describe the mitogenome sequences and phylogenetic positioning of *Oreochromis andersonii* and *O*. *macrochir* among the African cichlids. The complete mitochondrial DNA sequences were determined for *O*. *andersonii* and *O*. *macrochir*, two important aquaculture and fisheries species endemic to southern Africa. The complete mitogenome sequence lengths were 16642 bp and 16644 bp for *O*. *andersonii* and *O*. *macrochir* respectively. The general structural organization follows that of other teleost species with 13 protein–coding genes, 2 *rRNA*s, 22 *tRNA*s and a non-coding control region. Phylogenetic placement of the two species among other African cichlids was performed using Maximum Likelihood (ML) and Bayesian Markov-Chain-Monte-Carlo (MCMC). The consensus trees confirmed the relative positions of the two cichlid species with *O*. *andersonii* being very closely related to *O*. *mossambicus* and *O*. *macrochir* showing a close relation to both species. Among the 13 mitochondrial DNA protein coding genes *ND6* may have evolved more rapidly and *COIII* was the most conserved. There are signs that *ND6* may have been subjected to positive selection in order for these cichlid lineages to diversify and adapt to new environments. More work is needed to characterize the southern Africa cichlids as they are important species for capture fisheries, aquaculture development and understanding biogeographic history of African cichlids. Bio-conservation of some endangered cichlids is also essential due to the threat by invasive species.

## Introduction

Africa is the origin centre for cichlid diversity with well over 2000 species having diverse morphology, behaviour and ecology [1]. In Southern Africa *Oreochromis andersonii* (Castelnau 1861) and *Oreochromis macrochir* (Boulenger 1912) are two important mouth brooding endemic cichlid species in this region [2]. The former occurs in the upper Zambezi, Middle Zambezi, Kafue, Okavango and Cunene Rivers while the latter is distributed in the Upper Zambezi, Kafue, and Congo River systems [2–4]. *Oreochromis macrochir* (Green head or longfin tilapia) has further been introduced to the Hawaiian Islands, Okavango and Ngami region and Cunene River basin [4]. *Oreochromis andersonii* (Three-spot tilapia) and *O*. *macrochir* are important for both capture fisheries and aquaculture in Southern Africa [5]. However, due to the increase in fishing pressure as a result of an ever growing human population in this region as well as the introduction of Nile tilapia (*O*. *niloticus*) in almost all river systems where the native species occur, populations of these native species has greatly dwindled to vulnerable levels [6,7]. Nile tilapia hybridization with these native species and consequent decline in their population may as well make these species critically endangered in some Southern African rivers such as the Kafue River [8–10]. Despite Nile tilapia dominating aquaculture production in Southern Africa, efforts have been made to domesticate native species. This is partly as a result of the growing concern of ecosystem changes in most river systems due to Nile tilapia invasion. A number of studies in Zambia have shown potential for aquaculture of some native tilapia species [11–14]. In-fact, the Zambian Department of Fisheries have adopted *O*. *andersonii* as a candidate species for aquaculture development [15].

Molecular genetic studies on cichlids in Africa have focused primarily on phylogenetics of East African Lakes because of the spectacular adaptive radiation and explosive speciation in these lakes (for example, [16–19]). Nile tilapia has also received global attention because of its importance as an edible fish [20]. A few peer reviewed scientific papers have reported the use of molecular genetics in the study of endemic Southern African cichlids commercially important for fisheries and aquaculture. Phylogenetic relationships have been inferred among and between cichlid species based on selected mitochondrial DNA regions and some allozymes [21–26]. However, none of these phylogenetic analyses were based on complete mitogenome sequences.

Among the important cichlids for aquaculture in Southern Africa (*O*. *andersonii*, *O*. *macrochir*, *O*. *mossambicus*, *O*. *niloticus*, and *Tilapia rendalli*) complete mitogenome sequence have only been determined for *O*. *mossambicus* and *O*. *niloticus*.

In this study we describe the complete mitogenome sequences of *O*. *andersonii* and *O*. *macrochir* deposited in the GenBank with accession numbers MG603674 and MG603675 respectively. Based on the mitogenome sequences of these two species and other cichlids we confirm the position of these species among the cichlid species of Africa and analyse the evolutionary rates of the protein coding genes.

## Materials and methods

### Sample collection and DNA extraction

The Key Laboratory of Aquatic Genetic Resources and Utilization of Shanghai Ocean University approved the study protocol. All applicable international guidelines for the care and use of animals were followed. Three tissue samples (fin clips) each of *O*. *andersonii* and *O*. *macrochir* were collected from the Upper Zambezi River (16.10 S, 23.295 E) and Lake Bangweulu (11.35 S, 29.58 E) in Zambia respectively. This was conducted with approval from the Zambian Department of Fisheries. They were collected using gill nets and anaesthetized using clove oil (2–3 drops clove oil in a litre of water). The fin clips were obtained from each fish sample and preserved in absolute ethanol placed in 1.5 μL microtubules. These areas had no report of *O*. *niloticus* presence at the time of collecting the tissues. DNA was extracted from fin clips using a TIANamp Marine Animals DNA Kit using the manufacturer’s instructions (Tiangen, China).

### PCR amplification and sequencing

Thirty primers were designed for both *O*. *andersonii* and *O*. *macrochir* and 2 other primers for each species (S1 Table). The primers were designed using aligned complete mitogenomes of *O*. *mossambicus* (Accession number: AY597335.1) and *O*. *niloticus* (Accession number: GU370126.1). PCR was performed using an Eppendorf Thermal Cycler (Eppendorf, Germany). The total reaction mixture of 25.0 μl containing 17.5 μl distilled water, 4.5 μl PCR mix (Tiangen, China), 1.0 μl forward primer, 1.0 μl reverse primer, and 1.0 μl template DNA (50 ng/ μl) were used. The reaction was denatured at 94°C for 5 minutes, followed by 35 cycles of denaturation at 94°C for 30 seconds, annealing at 48–53°C for 30 seconds and elongation at 72°C for 45 seconds; the last extension step was carried out at 72°C for 7 minutes. Agarose (1.0%) electrophoresis was performed to visualize the PCR products. The PCR products were purified using the 3S Spin PCR product Purification Kit (Biocolor Inc., Shanghai, China). The purified DNA was sequenced on an ABI 3730 xl capillary sequencer employing the same primers as for PCR. The various regions of mitochondrial DNA were sequenced and projected as electronic outputs by the computer connected to the sequencer.

### Sequence editing, alignment and annotation

The raw sequences of the various regions of mitochondrial DNA of *O*. *andersonii* and *O*. *macrochir* were edited and assembled using BioEdit Version 7.2.6 [27]. They were further edited and aligned using complete mitogenomes of *O*. *niloticus* and *O*. *aureus* [28], *O*. *variabilis* [29] and *O*. *mossambicus* (accession number: AY597335.1). Blast database searches on NCBI site were performed to verify the target sequences amplified. Transfer RNA (*tRNA*) genes and their secondary structures were identified using tRNAScan-SE 2.0 [30]. MEGA Version 7.0.26 [31] was used to calculate the composition of amino acids, nucleotides and codon usage in the sequences. Annotation of the sequences were performed with DOGMA [32], MITOS [33], and MitoAnnotator [34]. Further verification was done with *O*. *niloticus* and *O*. *mossambicus* genome organization. The nucleotide composition skewness was measured following the formulas: AT skew [(A − T)/(A + T)] and GC skew [(G − C)/(G + C)] [35]. MEGA Version 7.0.26 was used to test for mode of selection in protein coding genes acting at non-synonymous sites using the ration non-synonymous/synonymous (*dN/dS)*.

### Phylogenetic analysis

To infer phylogeny, mitogenomes of 29 other cichlids mainly found in Southern Africa were obtained from the NCBI GenBank site (Table 1). The protein-coding genes were used for phylogenetic analysis (including the two novel genomes of *O*. *andersonii* and *O*. *macrochir*) except *ND6* which is encoded by the opposite strand and considered to possess a distinct heterogeneous base composition from the other 12 protein-coding genes [36]. The concatenated protein sequences were aligned using MUSCLE with default settings [37]. Furthermore, we analysed the phylogenetic relations of the same 30 cichlid species including the 2 novel sequences of *O*. *andersonii* and *O*. *macrochir* using a complete mitochondrial NADH dehydrogenase subunit 2 (*ND2*) gene sequences obtained from the NCBI GenBank. In the analysis using mitochondrial locus *ND2* we included another species *Coptodon rendalli*. The sequences were also aligned using MUSCLE with default settings performed in MEGA 7.0.26.

**Table 1:** List of species with their accession numbers used in phylogenetic analysis in this study.

Species	Accession No.	Reference	Species	Accession No.	Reference
*Oreochromis mossambicus*	AY597335.1	-	*Tylochromis polylepis*	NC_011171	[40]
*Oreochromis niloticus*	GU370126.1	[28]	*Haplochromis burtoni*	NC_027289	[41]
*Oreochromis aureus*	NC_013750	[28]	*Pundamilia nyererei*	NC_028011	[42]
*Oreochromis niloticus GIFT*	GU477624.1	-	*Copadichromis virginalis*	NC_029761	-
*Oreochromis variabilis*	NC_026109	[29]	*Fossorochromis rostratus*	NC_028089	[43]
*Oreochromis esculentus*	NC_025555	-	*Aulonocara stuartgranti*	NC_029380	[44]
*Coptodon zillii*	NC_026110	[29]	*Tropheus duboisi*	NC_009063	[45]
*Oreochromis* sp ‘red tilapia’	NC_014060	-	*Neolamprologus brichardi*	NC_009062.1	[45]
*Sarotherodon melanotheron*	NC_015611.1	[38]	*Petrochromis trewavasae*	NC_018814.1	[46]
*Serranochromis robustus*	NC_031418.1	-	*Buccochromis nototaenia*	NC_031416.1	
*Pseudotropheus crabro*	NC_018559.1	[39]	*Placidochromis longimanus*	NC_028156.1	-
*Petrotilapia nigra*	NC_018557.1	[39]	*Lethrinops lethrinus*	NC_031419.1	-
*Astatotilapia calliptera*	NC_018560.1	[39]	*Maylandia zebra*	NC_027944.1	
*Nimbochromis linni*	NC_018558	[39]	*Hemitilapia oxyrhyncha*	NC_031415.1	-
*Cynotilapia afra*	NC_018564	[39]			

Phylogenetic analysis was performed using Maximum Likelihood (ML) in MEGA Version 7.0.26 [31] and Bayesian Markov-Chain-Monte-Carlo (MCMC) method in MrBayes (Version 3.2.6) [47]

The optimal nucleotide substitution model selected was based on the General Time Reversible model (GTR + G + 1) obtained from a Model test in MEGA X [48]. It was based on a non-uniformity of evolutionary rates modelled by using a discrete Gamma distribution (+G) with 5 rate categories and by assuming that a certain fraction of the sites are evolutionary invariable (+1). Maximum Likelihood analysis was based on this General Time Reversible model with bootstrap consensus tree inferred from 1000 replicates taken to represent the evolutionary history of the taxa analysed. Branches corresponding to partitions reproduced in less than 50% bootstrap replicates were collapsed. Using the same nucleotide substitution model the Bayesian posterior probabilities were estimated using 1,000,000 generations, sampling every 100 generations at which point the standard deviation of split frequencies of the two independent runs was below 0.01. A consensus tree was constructed from the saved trees after discarding the first 25% trees as burn-in.

### Relative rate of protein coding gene evolution

To understand the measure of strength and mode of selection acting on protein coding genes we measured the ratio of non-synonymous to synonymous substitutions (*dN*/*dS*). This is because this ratio tends to differ over evolutionary time in protein coding genes. All 13 protein coding gene sequences were obtained from 20 different cichlid species and were aligned using a codon-based model in MUSCLE module of MEGA Version 7.0.26 [31]. Each gene was aligned separately. The rate of *dN*/*dS* was calculated using Maximum Likelihood computations conducted using HyPhy software package [49] in MEGA. Codon positions included were 1^st^ + 2^nd^ + 3^rd^ + Noncoding. All positions containing gaps and missing data were eliminated. The overall *p*-genetic distance calculation was performed on the 1^st^ and 2^nd^, 3^rd^ and whole sequence codon positions. Evolutionary analyses were conducted in MEGA X [48]

## Results and discussion

### Mitogenome annotation

Annotation results of *O*. *andersonii* and *O*. *macrochir* using three methods; DOGMA, MITOS and MitoAnnotator [32–34] revealed some differences in sizes of the protein coding genes and the two *rRNA*s. However, annotation results using the three methods were similar for all *tRNA*s (S2 Table). The largest difference was observed in gene *ND5* were DOGMA and MITOS differed from MitoAnnotator in the end position because of omission of stop codon bases in the annotation provided by MitoAnnotator (S2 Table). The gene sizes for *O*. *andersonii* and *O*. *macrochir* were similar in all the three annotation methods used except *ND2* final position with a difference of 7 bp and *ND5* initial position with a difference of 3 bp (S2 Table). Comparing the results obtained, annotation from Mitoannotator was selected for submission to the GenBank (Table 2).

**Table 2:** Complete automated annotated sequences of *O*. *andersonii* (16642 bp) and *O*. *macrochir* (16644 bp) generated in MitoAnnotator.

Protein	Position		Size (bp)	Codon			Strand^c^
	Start	Stop		Start	Stop^a^	Intergenic nucleotide^b^	H
tRNA^Phe^	1	69	69				H
12S rRNA	70	1013	944			0	H
tRNA^Val^	1014	1085	72			0	H
16S rRNA	1086	2779	1694			0	H
tRNA^Leu^	2780	2853	74			0	H
ND1	2854	3828	975	ATG	TAG	0	H
tRNA^Ile^	3832	3901	70			3	H
tRNA^Gln^	3901	3971	71			-1	L
tRNA^Met^	3971	4039	69			0	H
ND2	4040	5094	1055	ATG	TAA	0	H
tRNA^Trp^	5095	5166	72			0	H
tRNA^Ala^	5168	5236	69			1	L
tRNA^Asn^	5238	5310	73			1	L
tRNA^Cys^	5344	5409	66			33	L
tRNA^Tyr^	5410	5479	70			0	L
COI	5481	7082	1602	GTG	TAA	1	H
tRNA^Ser^	7083	7153	71			0	L
tRNA^Asp^	7157	7229	73			3	H
COII	7235	7925	691	ATG	T++	5	H
tRNA^Lys^	7926	7999	74			0	H
ATPase 8	8001	8168	168	ATG	TAA	1	H
ATPase 6	8159	8832	674	ATG	TAA	-10	H
COIII	8833	9616	784	ATG	TAA	0	H
tRNA^Gly^	9617	9688	72			0	H
ND3	9689	10037	349	ATG	TAG	0	H
tRNA^Arg^	10038	10106	69			0	H
ND4L	10107	10403	297	ATG	TAA	0	H
ND4	10397	11786	1390	ATG	T++	-7	H
tRNA^His^	11787	11855	69			0	H
tRNA^Ser^	11856	11922	67			0	H
tRNA^Leu^	11927	11999	73			4	H
ND5	12000	14300	2301	ATG	TAA	0	H
ND6	13840	14361	522	ATG	TAA	-461	L
tRNA^Glu^	14362	14430	69			0	L
Cyt b	14435	15575	1141	ATG	T++	4	H
tRNA^Thr^	15576	15647	72			0	H
tRNA^Pro^	15648	15717	70			0	L
CR	15718	16642/16644	925/927			0	-

The forward slashes (/) denote the values of *O*. *andersonii*/ *O*. *macrochir*, if none the values for both species are identical.

^a^ T++ represent incomplete stop codon.

^b^The positive numbers indicate nucleotides separating two adjacent genes while the negative numbers represent nucleotide overlap.

^**c**^ H = heavy and L = light strands.

### Mitogenome organization

**Fig 1:**
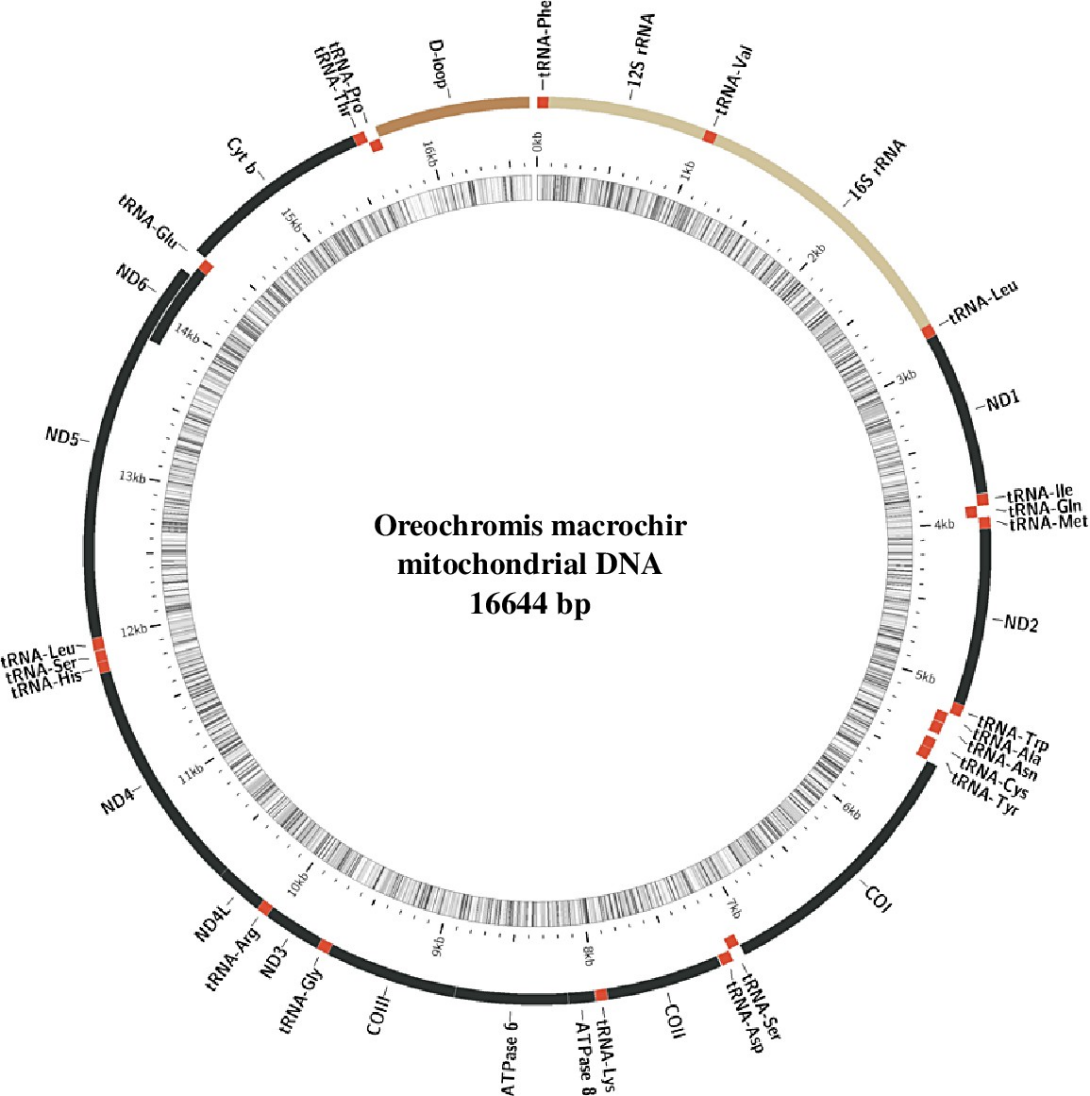
Mitogenome organisation of *O*. *macrochir* generated by MitoAnnotator. The genes on the outer side of the circle are coded on the H-strand while those on the inner circle are coded with L-strand.

The complete mitogenome sequence size of *O*. *andersonii* and *O*. *macrochir* were 16642 bp (accession #: MG603674) and 16644 bp (accession #: MG603675) respectively. This was similar to the published mitogenomes sequences of other *Oreochromis* species i.e. *O*. *niloticus* with 16625 bp and *O*. *aureus* with 16628 bp [28]; *Oreochromis variabilis* with 16626 bp [29] and GenBank deposited sequence of *O*. *mossambicus* (AY597335.1). As anticipated, the general structural organization follows those of other teleost species with 13 protein coding genes, 2 *rRNA*s, 22 *tRNA*s, and a non-coding control region (Table 2 and Figs 1 and S1). Both species were observed to exhibit the Heavy (H) and Light (L) strand coding pattern previously observed in other teleosts. The L-strand was observed in one protein coding gene (*NADH* dehydrogenase subunit 6 (*ND6*)) and eight *tRNA*s (*tRNA*^*Gln*^, *tRNA*^*Ala*^, *tRNA*^*Asn*^, *tRNA*^*Cys*^, *tRNA*^*Tyr*^, *tRNA*^*Ser*^, *tRNA*^*Glu*^, *tRNA*^*Pro*^). The start codons for protein coding genes for both *O*. *andersonii* and *O*. *macrochir* were ATG except for gene *CO1* which was GTG. The stop codons on the other hand were either TAG or TAA except for gene *CO11*, *ND4*, and *Cyt b* which had incomplete stop codons of T++. The incomplete codon stops observed for the two species is a common feature in most vertebrates including some fish species [50–52].

The overall nucleotide base composition was very similar between the two species. For *O*. *andersonii*; T = 26.3%, C = 30.1%, A = 28.0%, G = 15.6% whereas *O*. *macrochir* T = 26.2%, C = 30.2%, A = 28.1%, G = 15.5%. Further, the GC% and AT% were the same for both species at 45.7 and 54.3 respectively (Table 3). This base composition is very similar to what has been reported for other *Oreochromis* species [28, 29] and other species as well [29,53]. The lower value for GC compared to AT is another common feature which has been observed in most vertebrate mitogenome resulting from anti-bias against G in the third codon position. The species in this study had lower values of G in all the three codon positions of protein coding genes and non-coding control region (Table 3). Furthermore, the GC-skew and AT-skew which describe the overall patterns of nucleotide composition in DNA sequences [35], were -0.317 and 0.031 for *O*. *andersonii* and -0.322 and 0.035 for *O*. *macrochir* respectively. This result shows an excess of C over G and excess of A over T on the heavy strand (Table 3).

**Table 3:** Base percent composition of complete mitogenome sequences of *O*. *andersonii* (16642 bp) and *O*. *macrochir* (16644 bp) generated in MEGA 7.0.26.

		Complete mitogenome		Protein coding genes		ND6		tRNAs		rRNAs		Control Region	
Codon position	% Nucleotide composition	*O.*A*	*O.*M*	*O*.*A*	*O*.*M*	*O*.*A*	*O*.*M*	*O*.*A*	*O*.*M*	*O*.*A*	*O*.*M*	*O*.*A*	*O*.*M*
1	T	27.0	27.0	31.0	31.0	35.0	35.0	26.0	26.0	19.0	19.0	32.0	31.0
	C	31.3	31.3	30.1	30.1	8.0	8.0	22.8	23.0	26.6	26.7	20.4	19.1
	A	27.7	27.9	25.3	25.2	6.9	6.9	27.2	27.2	32.4	32.6	32.0	34.3
	G	13.8	13.8	13.9	14.0	50.0	50.0	23.6	23.4	21.7	21.7	15.5	15.5
2	T	24.0	24.0	27.0	27.0	43.0	43.0	31.0	31.0	21.0	22.0	31.0	30.0
	C	29.8	30.0	36.2	36.2	21.8	22.4	20.7	20.7	25.7	25.6	20.5	23.6
	A	29.3	29.3	26.6	26.8	10.9	10.9	29.0	29.0	32.5	32.7	32.5	32.7
	G	16.5	16.4	10.0	9.9	24.1	24.1	19.9	19.9	20.4	20.3	15.6	13.6
3	T	27.0	27.0	24.0	24.0	37.0	37.0	24.0	24.0	21.0	21.0	35.0	38.0
	C	29.3	29.2	30.8	31.0	1.1	1.1	21.2	21.0	28.0	28.2	20.8	19.4
	A	27.0	26.9	26.9	26.8	20.5	19.4	28.4	28.6	31.9	31.9	31.2	29.4
	G	16.3	16.3	17.9	17.9	41.4	42.5	26.4	26.3	19.5	19.2	13.0	13.3
Total compositi-on	T(U)	26.3	26.2	27.5	27.4	38.3	38.3	27.0	27.0	20.5	20.4	32.9	33.0
	C	30.1	30.2	32.4	32.5	10.3	10.5	21.6	21.6	26.8	26.8	20.5	20.7
	A	28.0	28.1	26.2	26.2	12.8	12.3	28.2	28.2	32.3	32.4	31.9	32.1
	G	15.6	15.5	13.9	13.9	38.5	38.9	23.2	23.3	20.5	20.4	14.7	14.1
	% GC	45.7	45.7	46.3	46.4	48.9	49.4	44.7	44.9	47.3	47.2	35.2	34.8
	% AT	54.3	54.3	53.7	53.6	51.1	50.6	55.3	55.1	52.7	52.8	64.8	65.2
	GC Skew	-0.317	-0.322	-0.400	-0.401	0.578	0.575	0.036	0.038	-0.133	-0.136	-0.165	-0.190
	AT Skew	0.031	0.035	-0.024	-0.022	-0.499	-0.514	0.022	0.022	0.223	0.227	-0.015	-0.014
	Total length (bp)	16642	16644	11427	11427	522	522	1554	1554	2638	2638	925	927

*O.*A* = *Oreochromis andersonii*

*O.*M* = *Oreochromis macrochir*

Intergenic overlaps were observed in 4 genes and spacers in 10 genes in the mitogenome of *O*. *andersonii* and *O*. *macrochir*. The most significant overlap of 461 nucleotides was observed between *ND5* and *ND6*. This large overlap is not a common feature among most reported teleost mitogenomes. Overlap of 10 nucleotides between *ATPase 8* and *ATPase 6* was also observed for both species followed by *ND4L* and *ND4* gene with overlap of 7 nucleotides. Intergenic spacers totalled 56 bp in 10 regions for both *O*. *andersonii* and *O*. *macrochir* (see Table 2). The most significant intergenic spacers in both species were between *tRNA*^*Asn*^ and *tRNA*^*Cys*^ (33 nucleotides) followed by *tRNA*^*Asp*^ and *COII* (5 nucleotides). Again intergenic overlaps and spacers follow what has been reported for most vertebrate mitogenomes inclusive of different fish species except for the overlap between *ND5* and *ND6*.

### Protein coding genes

The nucleotide composition of the protein coding genes was very similar between *O*. *andersonii* and *O*. *macrochir* (Table 3). The total GC% composition was 46.3 and AT% was 53.7 for *O*. *andersonii* and 46.4 and 53.6 for *O*. *macrochir*. The GC-skew for *O*. *andersonii* and *O*. *macrochir* were -0.400 and -0.401 and the AT-skew values were -0.024 and -0.022 respectively. However, the *L*- strand gene *ND6* had a positive GC-skew value for both species (Table 3). Again it showed overall anti-G bias supporting earlier findings in fish mitogenomes [50,52,54]. However, unlike many authors who report strong anti-G bias on third codon positions, both *O*. *andersonii* and *O*. *macrochir* were observed to have the strong anti-G bias on the second codon position (*O*. *andersonii* = 10.0, *O*. *macrochir* = 9.9).

**Fig 2:**
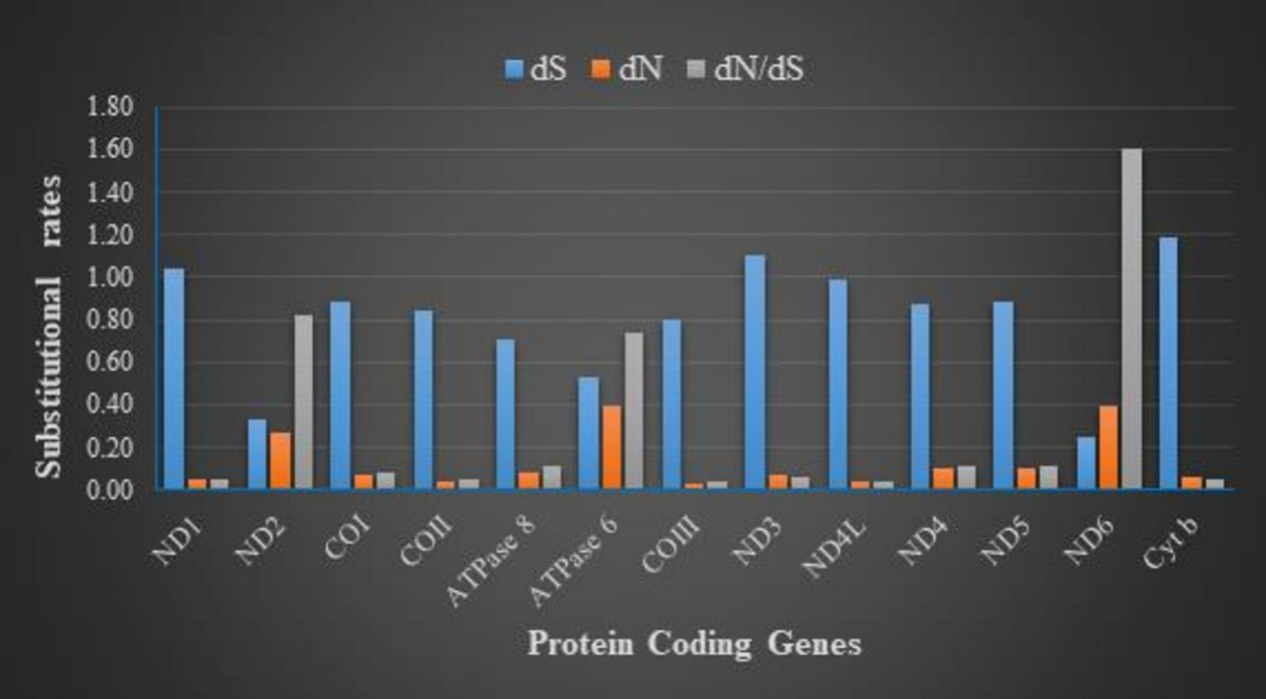
The rate of non-synonymous substitution (*dS*), rate of synonymous substitution (*dN*) and the ratio of *dS* and *dN* for each protein coding gene.

The adaptive radiation of African cichlids is unparalleled so far among the vertebrates. To understand the role of mitogenome protein coding genes in the evolution of these cichlid species we analysed the rate of non-synonymous (*dN*) and synonymous (*dS*) nucleotide substitutions in 13 protein coding genes of 20 species. These included; Riverine, Lake Victoria, Lake Tanganyika and Lake Malawi species (*Sarotherodon melanotheron*, *Coptodon zillii*, *Oreochromis aureus*, *O*. *niloticus*, *O*. *niloticus* GIFT, *O*. *mossambicus*, *O*. *variabilis*, *O*. *andersonii*, *O*. *macrochir*, *O*. *esculentus*, *Oreochromi*s sp ‘red tilapia’, *Petrochromis trewavasae*, *Tropheus duboisi*, *Astatotilapia calliptera*, *Cynotilapia afra*, *Maylandia zebra*, *Pundamilia nyererei*, *Tylochromis polylepis*, *Haplochromis burtoni*, *Lethrinops lethrinus*). The mean value of *dN* and *dN/dS* was highest in *ND6* gene and lowest in *COIII* gene for all the 13 protein coding genes (Fig 2). Our findings indicate that all protein coding genes evolved under purifying selection except for *ND6* gene which had an elevated rate of *dN*/*dS* of 1.860 indicative of evolution under positive selection. This indicates that various functional genes evolved under natural selection while *ND6* gene shows signs of evolution by mutation and genetic drift. Since mitochondrial genome is involved in the production of ATP which provides energy for cellular activities and other cellular processes, it is possible that geographical isolation may have affected the nucleotide substitutions in the mitochondrial protein coding genes. We can conclude that *ND6* gene may have evolved more rapidly than any other protein coding gene among these African cichlids.

**Fig 3:**
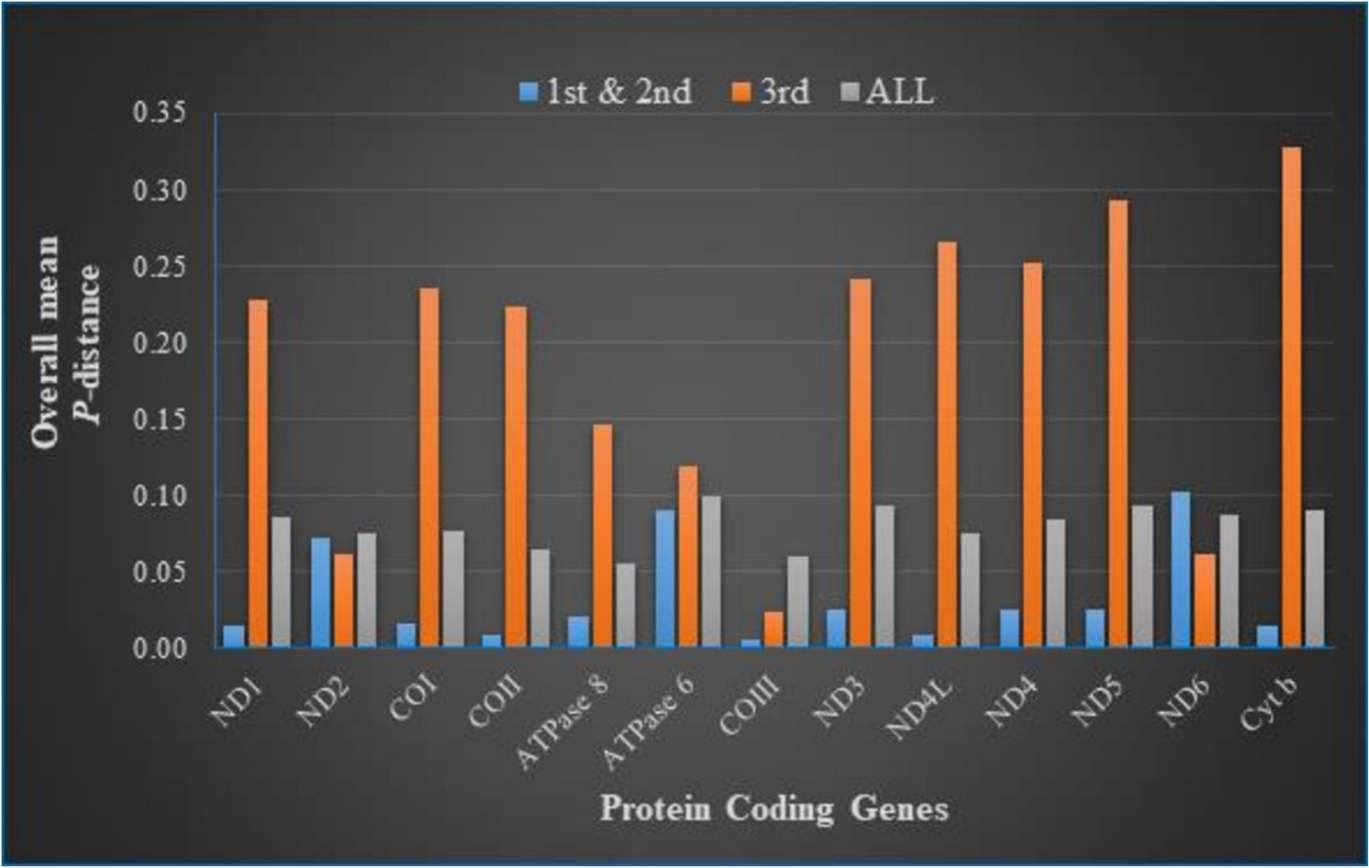
Estimates of average evolutionary divergence over all sequence pairs of 20 cichlid species for each of the 13 protein coding genes calculated based on codon positions 1^st^ + 2^nd^, 3^rd^ and full sequence.

The overall *p*-genetic distance was used to measure the conservation of the protein coding genes in the mitogenome of these 20 cichlid species. Calculations were performed on the 1^st^ and 2^nd^, 3^rd^ and whole sequence codon positions. On the 1^st^ and 2^nd^ codon positions, the highest overall mean *p*-distance was on gene *ND6* (0.1581) followed by *ATPase* 6 (0.1268) and least was gene *COIII* (0.0193). For the whole sequence, the highest overall *p*-distance was recorded in *ATPase* 6 (0.1389) followed by *ND6* (0.1288) and *ATPase* 8 (0.0990) had the least value (Fig 3). Based on these results *ND6* likely may have the highest evolutionary rate and *COIII* been the most conserved gene among the mitogenome protein coding genes of these cichlid species which could have radiated into many species due to geographical isolation [1].

Evidence of positive selection, though not very common, has been observed in some fishes [55,56], in horses [57,58] and in insects [59]. Other studies have shown the important role the mitogenome plays in adapting to new environments as reviewed in Castellana et al. 2011 [60]. The spectacular radiations of the East African rift valley Lakes Malawi and Tanganyika, Lake Victoria and surrounding smaller lakes and rivers may have driven the fixations of mutations in the mitogenome in such a way that the mitochondrial functions are guaranteed. This adaptation of some genes in the mitogenome involved in energy production could perhaps be exploited in aquaculture. The use of indigenous or autochthonous species may prove advantageous because they may have been selected to adapt to local environmental conditions.

### Ribosomal and transfer RNAs

The *tRNA* genes for both *O*. *andersonii* and *O*. *macrochir* possessed anti-codons that match the vertebrate mitochondrial code. The average length of the *tRNA*s for both species ranged between 66–74 bp while the total length was 1554 bp for both *O*. *andersonii* and *O*. *macrochir*. For both species the^*)*^ inferred secondary structures for all the 22 *tRNA*s, with exception of *tRNA*^*Ser*(*GCT*)^, folded into cloverleaf model. The common features of the secondary structures were: a 7 bp aminoacyl stem and anticodon loop, 5 bp TΨC and anticodon arms, and 4 bp DHU arm (S2 Fig). Similar results have been reported on South American catfish and Asian arowana [52,54]. However, non-complementary pairing and size variations in the secondary structures were observed in both species.

The two *rRNA*s for both species had a total length of 944 bp and 1694 bp which was within the range reported for vertebrate mitogenomes. The nucleotides percent compositions of *rRNA*s of *O*. *andersonii* (T = 20.5, C = 26.8, A = 32.3, G = 20.5) and *O*. *macrochir* (T = 20.4, C = 26.8, A = 32.4, G = 20.4) were very similar. They also showed a higher percentage of AT than GC pairs (52.7, 47.3 for *O*. *andersonii*; 52.8, 47.2 for *O*. *macrochir*) (Table 3)

### Non-coding region

The mitochondrial non-coding region or control region of *O*. *andersonii* and *O*. *macrochir* was located between *tRNA*^*pro*^ and *tRNA*^*phe*^ typical for vertebrate mitogenomes. Its length was 925 bp for *O*. *andersonii* and 927 bp for *O*. *macrochir*. The nucleotide base composition was similar between the two species although more variable compared to other regions of the mitogenome (Table 3). The AT% (35.2:34.8, *O*. *andersonii*: *O*. *macrochir*) composition to GC% (64.8:65.2, *O*. *andersonii*: *O*. *macrochir*) was also more highly skewed towards AT compared to other regions of the mitogenome due to anti-G bias on the third codon position.

**Fig 4:**
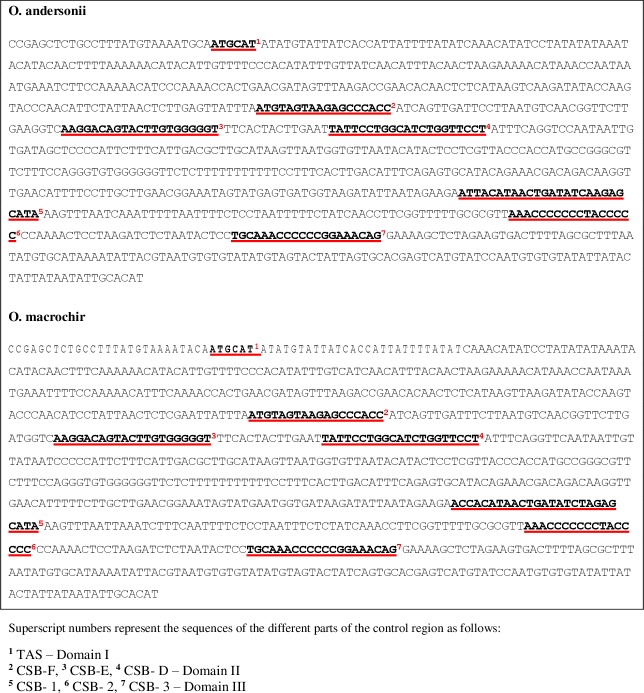
Different parts of the control region of *O*. *andersonii* and *O*. *macrochir*.

*Oreochromis andersonii* and *O*. *macrochir* control regions were aligned with four other species from the same genus to characterize the control region. Domain 1 consisted of a hypervariable region with a length of 281 bp for both species including a Termination -Associated Sequence (TAS) with motif- ATGCAT similar to a putative TAS of *O*. *aureus* [28]. Domain II or central conserved region was identified with three conservative sequence blocks (CSB) which are involved in heavy-H strand replication [61]. The first was CSB-F (ATGTAGTAAGAGCCCACC) followed by CSB-E (AAGGACAGTACTTGTGGGGGT) and then CBS-D (TATTCCTGGCATCTGGTTCCT) to complete domain II for both species. The third domain at the 3' end of the control region consisted of CBS-1 (*O*. *andersonii—*ATTACATAACTGATATCAAGAGCATA; *O*. *macrochir*- ACCACATAACTGATATCTAGAGCATA) and CBS-2 (AAACCCCCCCTACCCCC). The last conservative block observed was CBS-3 (TGCAAACCCCCCGGAAACAG) (Fig 4)

### Amino acid composition

**Fig 5:**
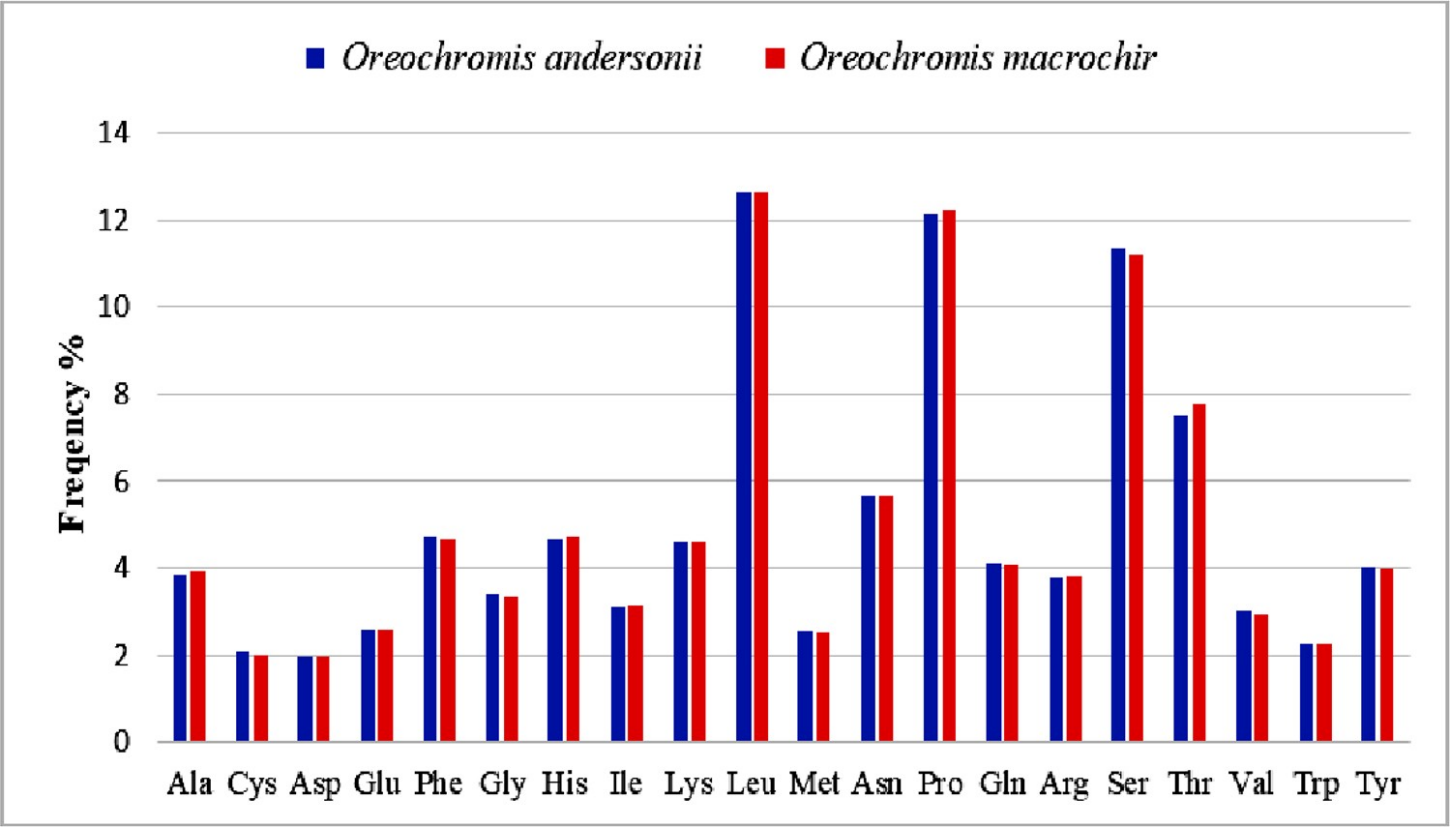
Amino acid frequency in the complete mitogenome of *O*. *andersonii* and *O*. *macrochir*.

For both *O*. *andersonii* and *O*. *macrochir* leucine, proline, serine, threonine, asparagine were the most frequently translated amino acids from the mitochondrial genome (Fig 5 and S3 Table). This is similar to the findings on other fish species [52,54]. The relative synonymous codon usage (RSCU) indicated that the most frequently used codon in the mitogenome of the two species was GCC for alanine with *O*. *andersonii* having an RSCU value of 1.74 while *O*. *macrochir* had 1.67. The second highest codon usage values were for serine (UCU) for *O*. *andersonii* having RSCU = 1.54 and Arginine (CGC) for *O*. *macrochir* having an RSCU = 1.58. RSCU values of 0.40 and 0.43 were observed to be the least for alanine (GCG) for *O*. *andersonii* and *macrochir* respectively.

### Phylogenetic analysis

**Fig 6:**
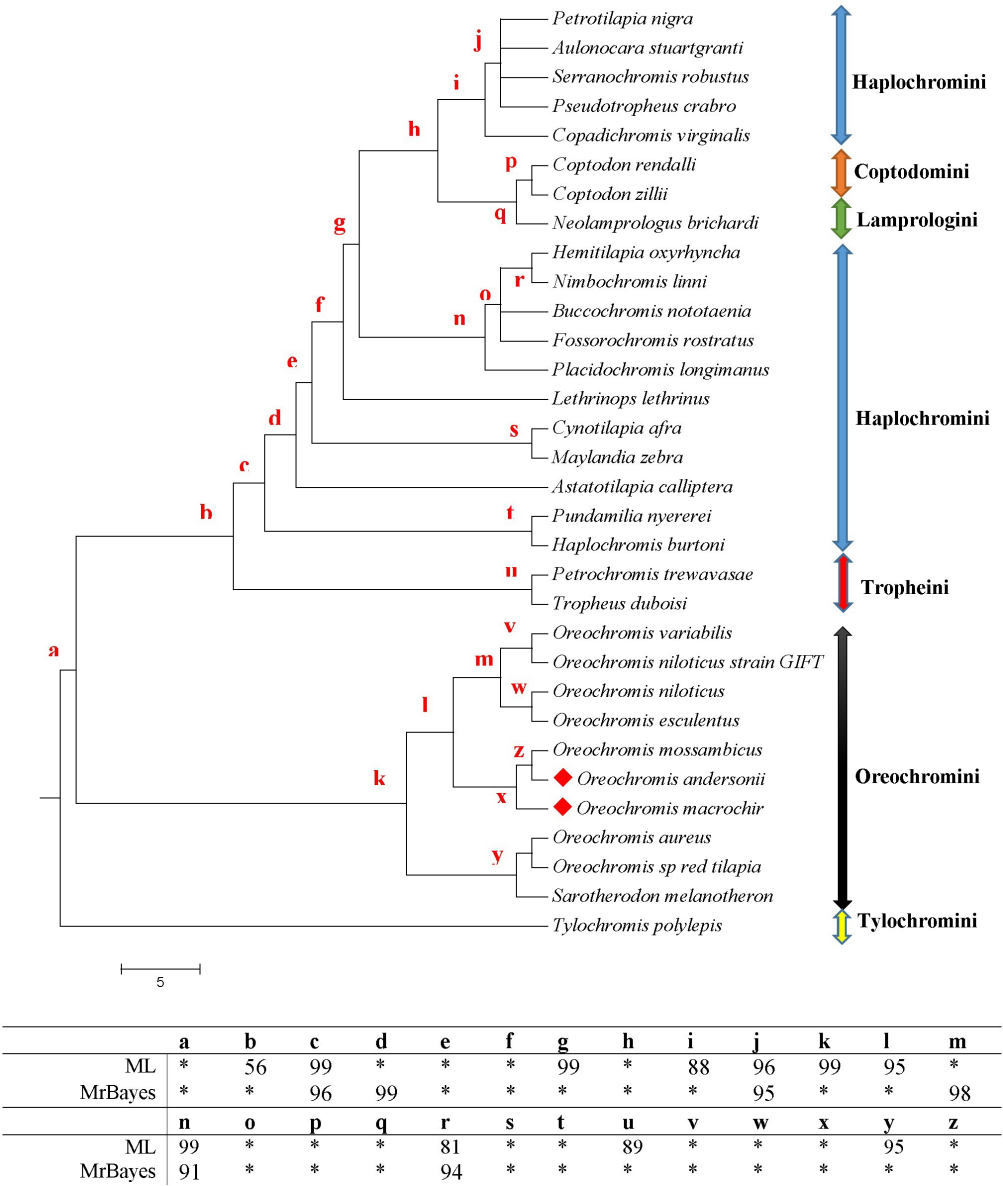
A phylogenetic tree of African cichlids showing the relative positions of *O*. *andersonii* and *O*. *macrochir* based on protein-coding gene *ND2*. The red letters ‘a’ to ‘z’ indicate the Bayesian posterior probability and maximum likelihood values for each node, while the asterisk ‘*’ indicate 100% value.

**Fig 7:**
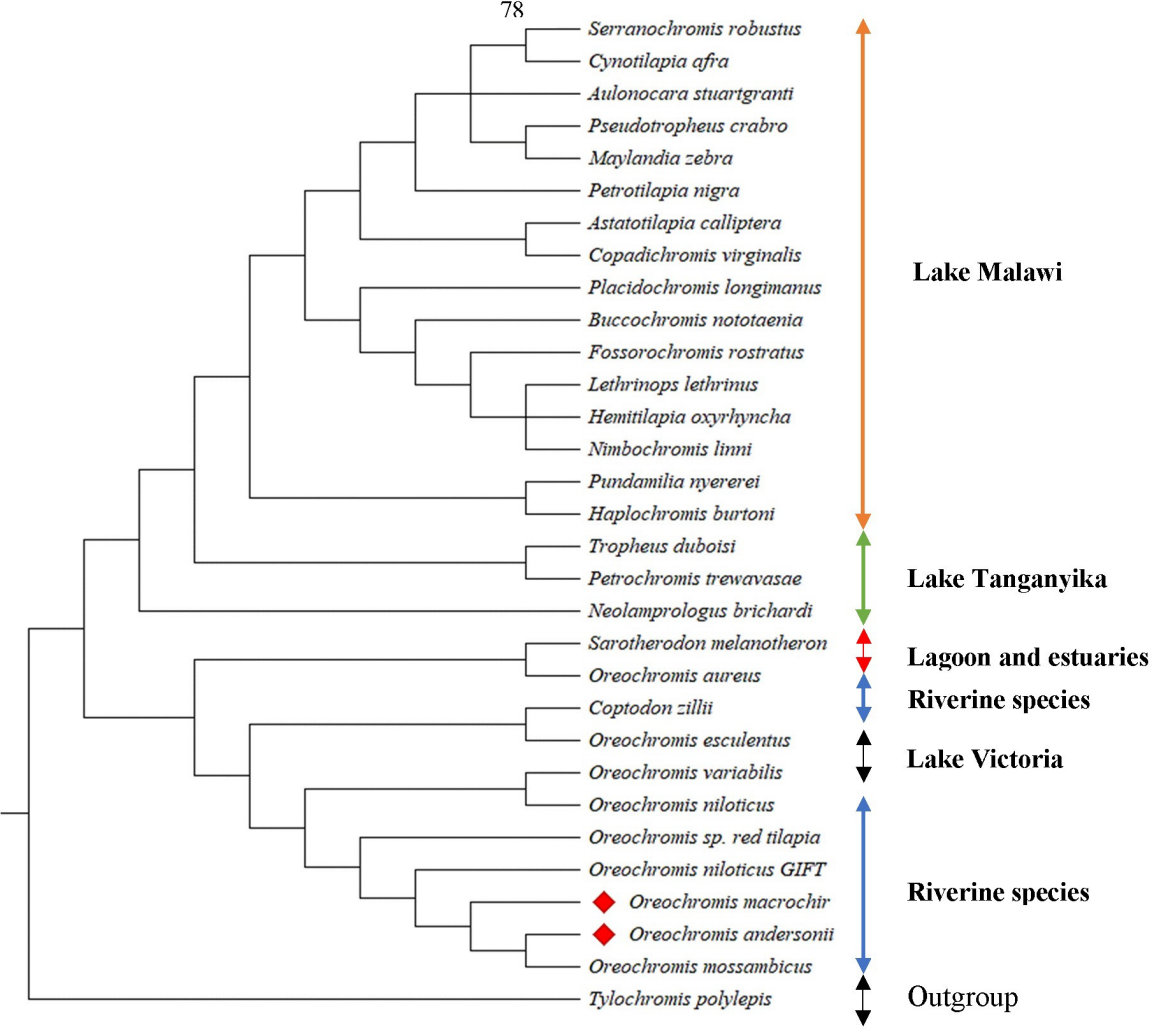
The phylogenetic tree showing the position of *O*. *andersonii* and *O*. *macrochir* with 29 other cichlid species based on Bayesian posterior probabilities of 12 concatenated protein genes (without *ND6*). All the node values not indicated recorded a 100% Bayesian posterior probability.

Phylogenetic analysis using 12 concatenated protein coding genes and another analysis using a mitochondrial locus *ND2* from 29 cichlid species were used to examine the phylogenetic placement of *O*. *andersonii* and *O*. *macrochir* among the cichlids of Africa. The consensus tree separates the species into clear clades or tribes; Haplochromini, Coptodomini, Lamprologini, Tropheini, Oreochromini and Tylochromini. This is consistent with recent classifications of the haplotilapiine cichlids of Africa [62] (Figs 6 and S3). However, the 12 concatenated protein coding gene sequences grouped *Coptodon zillii* (Coptodoini) with species from the Oreochromini tribe rendering the clade paraphyletic (Fig 7). This may probably have been due to misidentification of *C*. *zillii* because the other species clearly separate into their clades or tribes. The close relationship of *Sarotherodon melanotheron* to *Oreochromis species* rather forming a monophyletic clade is not yet certain and has been questioned by some authors such as Klett and Meyer [63] and previous authors even suggested the need to redefine the genus [64,65].

The three *Oreochromis* species (*O*. *macrochir*, *O*. *andersonii* and *O*. *mossambicus*) from Southern Africa in this phylogenetic tree seem to have evolved later compared to the west and east African species. Data from the consensus tree in this study placed *O*. *andersonii* closely related to *O*. *mossambicus* (Figs 6 and 7). This is in agreement with other previous phylogenetic trees [21, 63]. The close relatedness of these two species though not sharing the same habitat may indicate that they may have had separated most recently. On the other hand, *O*. *macrochir* clusters with both *O*. *mossambicus* and *O*. *andersonii* indicative of a closer phylogenetic relationship among these species of Southern Africa.

## Conclusion

This study has determined the complete mitochondrial DNA sequences of *O*. *andersonii* (16623 bp) and *O*. *macrochir* (16624 bp) and further characterization has confirmed their similarity to vertebrate mitochondrial DNA. Using Bayesian posterior probabilities and Maximum likelihood analysis of concatenated mitochondrial genome of 12 protein coding genes and *ND2* gene, consensus trees have revealed a close phylogenetic relationship between *O*. *andersonii* and *O*. *mossambicus*. Further, *O*. *macrochir* was found to be closely related to both *O*. *andersonii* and *O*. *mossambicus*. The protein coding genes evolved under purifying selection except for *ND6* which indicated evolution under positive selection. Gene *ND6* likely may have the highest evolutionary rate and *COIII* been the most conserved gene. This may indicate that there was a high adaptation level at the *ND6* gene in response to changing environments among these African cichlid species. More work is needed to characterize the southern Africa cichlids as they are important species especially for capture fisheries, aquaculture development and understanding biogeographic history of African cichlids.

## Supporting information

S1 Fig Mitogenome organisation of *O*. *andersonii* generated by MitoAnnotator. The genes on the outer side of the circle are coded on the H-strand while those on the inner circle are coded with L-strand.
(PDF)

S2 Fig Secondary structures of 22 tRNAs of mitochondrial genome of O. andersonii (A) and O. macrochir (B) generated by tRNAScan-SE 2.0
(PDF)

S3 Fig The phylogenetic tree showing the relative positions of *O*. *andersonii* and *O*. *macrochir* among 29 African cichlids based on Maximum Likelihood bootstrap consensus tree inferred from 1000 replicates of ND2 gene. (PDF)

S1 Table Designed primers used for amplification and sequencing of complete mitogenome of *O*. *andersonii* and *O*. *macrochir*. (PDF)

S2 Table Complete automated annotation of *O*. *andersonii* and *O*. *macrochir* mitochondrial genome. (PDF)

S3 Table Codon usage of complete mitogenome sequences of Oreochromis andersonii (16642 bp) and O. macrochir (16644 bp). (PDF)
