# Supplementary material for: Mitochondrial genome annotation and phylogenetic placement of Oreochromis andersonii and O. macrochir among the cichlids of southern Africa
Source: PLoS One. 2018 Nov 27;13(11):e0203095. doi: 10.1371/journal.pone.0203095 (PMC6258479; doi:10.1371/journal.pone.0203095)
Supplement: S1 Fig — The genes on the outer side of the circle are coded on the H-strand while those on the inner circle are coded with L-strand. (PDF) [file pone.0203095.s001.pdf]

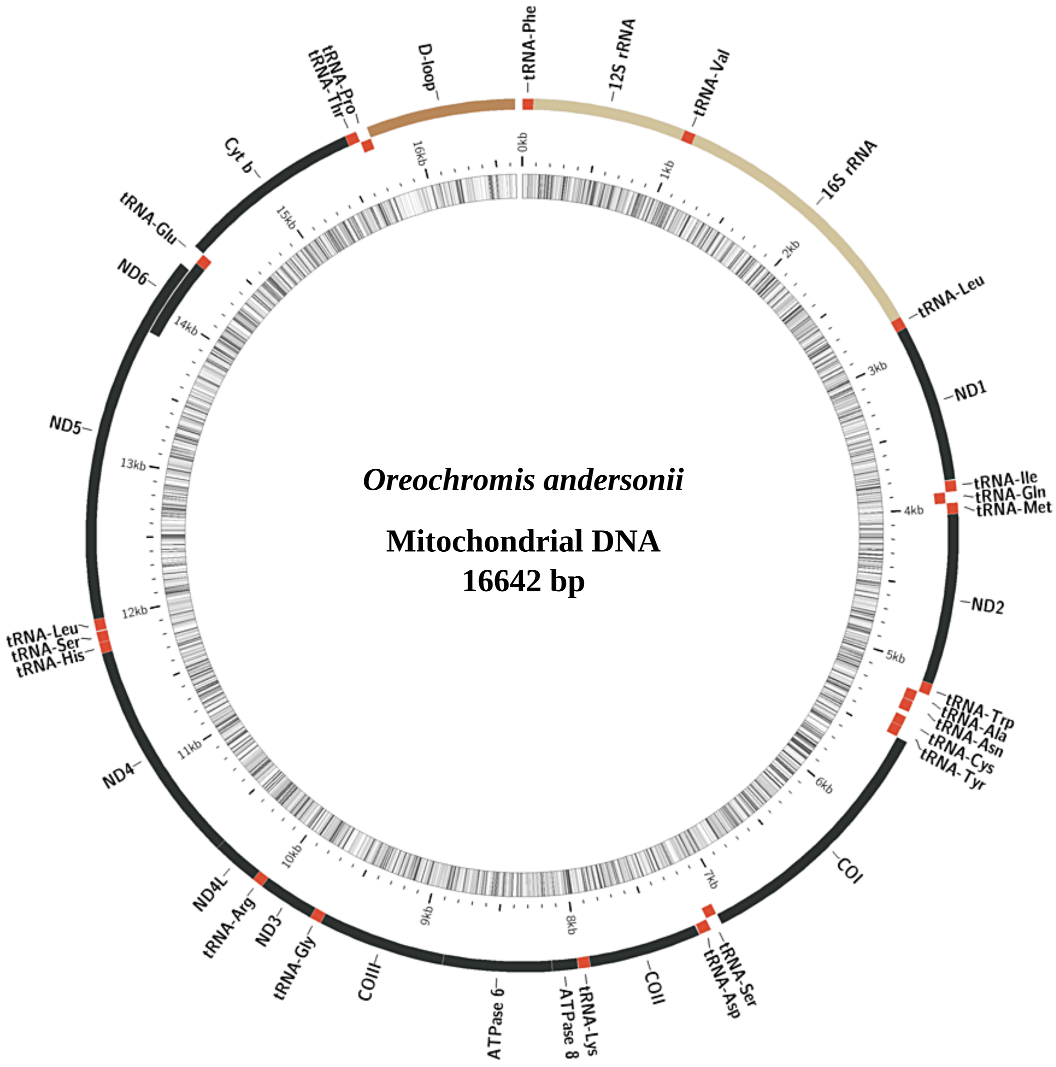

S1 Fig. Mitogenome organisation of *O. andersonii* generated by MitoAnnotator. The genes on the outer side of the circle are coded on the H-strand while those on the inner circle are coded with L-strand.
