# Supplementary material for: Mitochondrial genome annotation and phylogenetic placement of Oreochromis andersonii and O. macrochir among the cichlids of southern Africa
Source: PLoS One. 2018 Nov 27;13(11):e0203095. doi: 10.1371/journal.pone.0203095 (PMC6258479; doi:10.1371/journal.pone.0203095)
Supplement: S2 Fig — Secondary structures of 22 tRNAs of mitochondrial genome of O. andersonii (A) and O. macrochir (B) generated by tRNAScan-SE 2.0 (PDF) [file pone.0203095.s002.pdf]

(A)

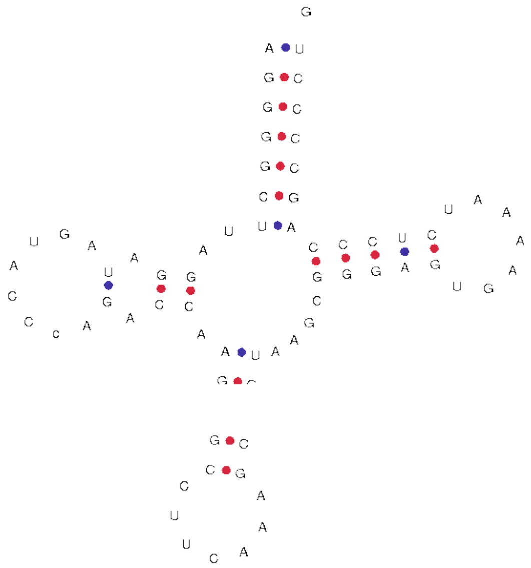

Tryotophan (TCA)

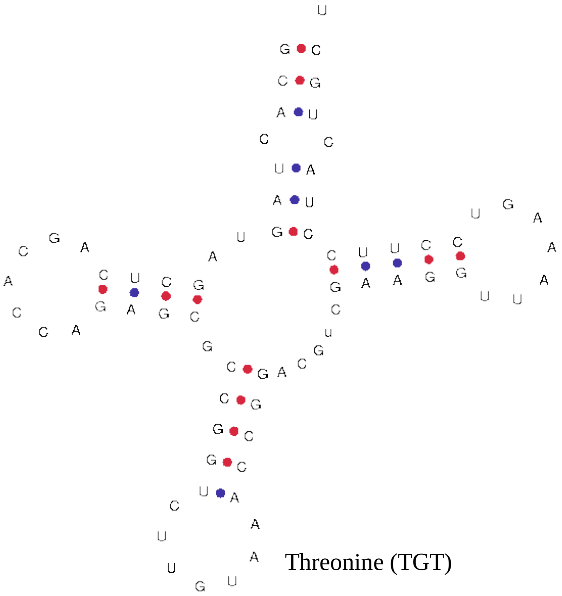

Threonine (TGT)

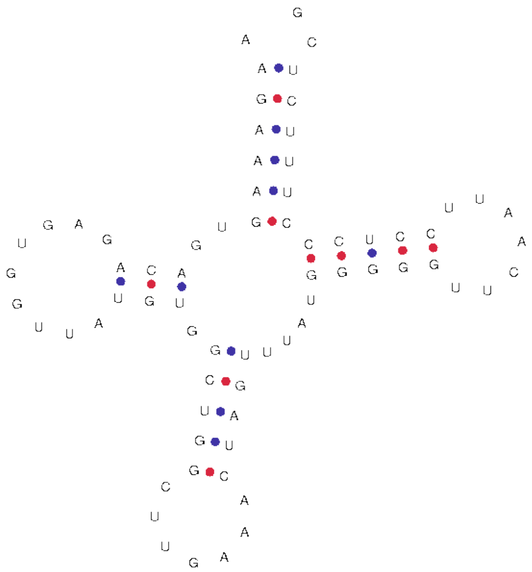

Serine (TGA)

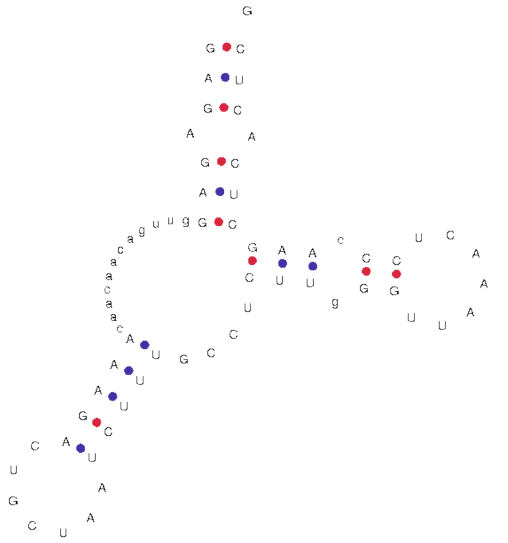

Serine (GCT)

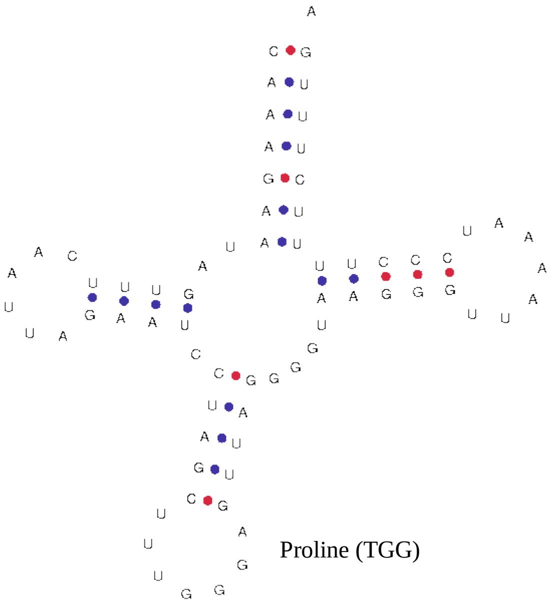

Proline (TGG)

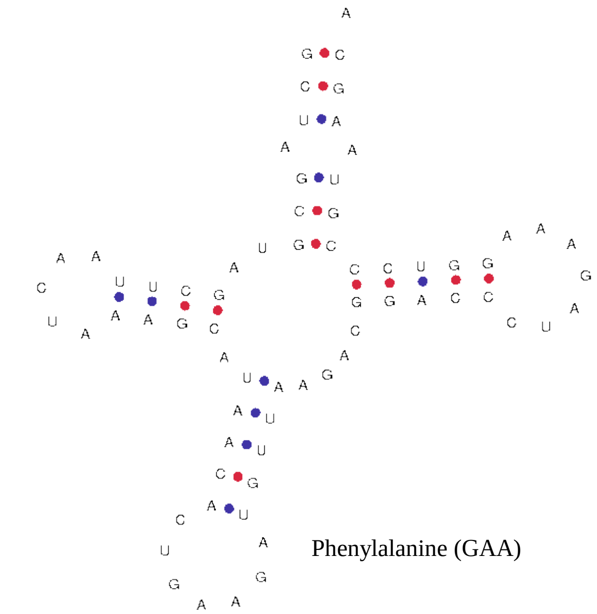

Phenylalanine (GAA)

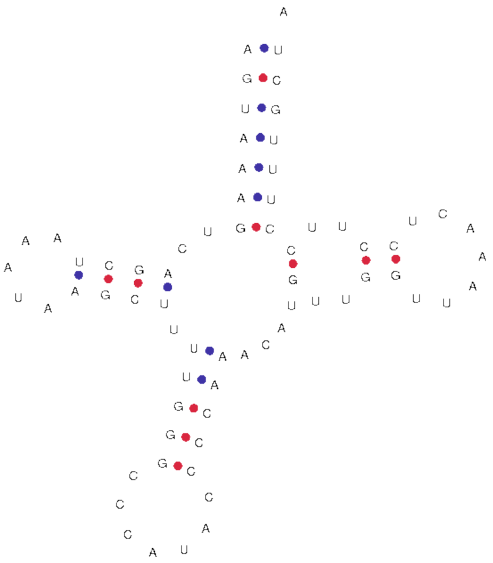

Methionine (CAT)

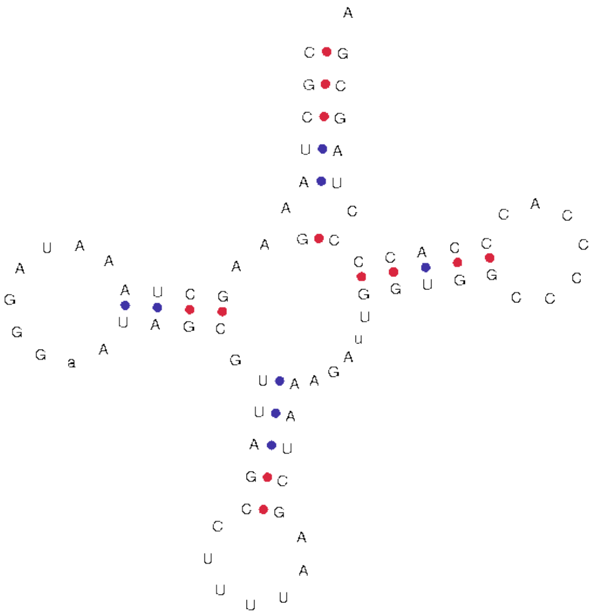

Lysine (TTT)

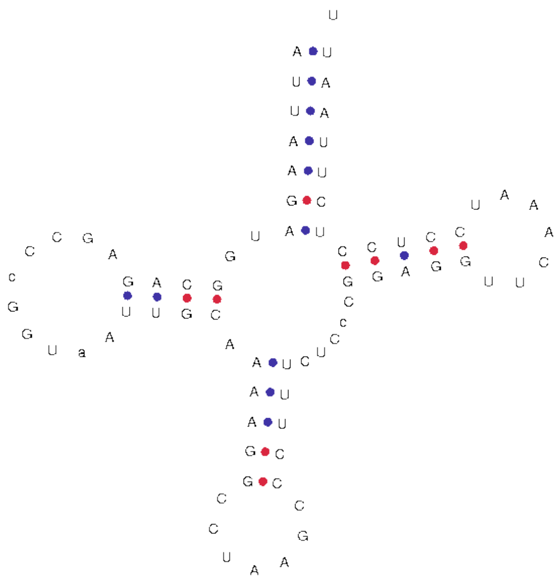

Leucine (TAA)

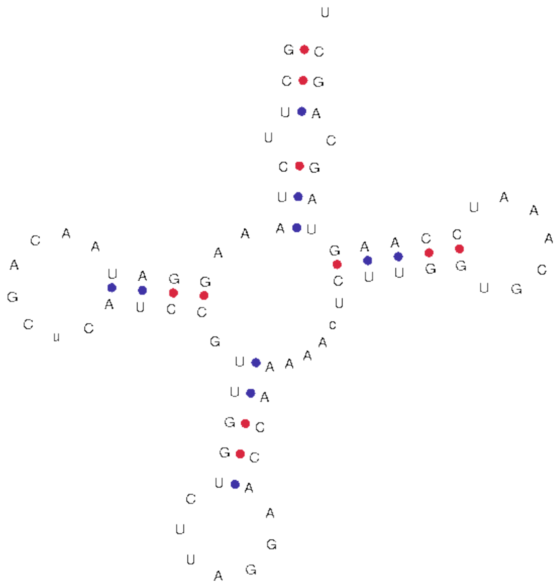

Leucine (TAG)

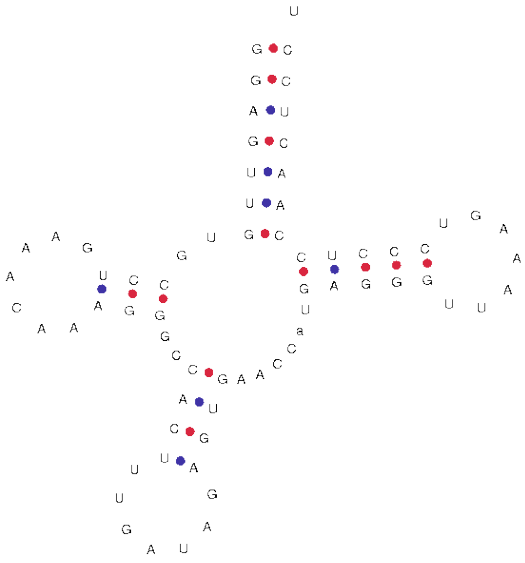

Isoleucine (GAT)

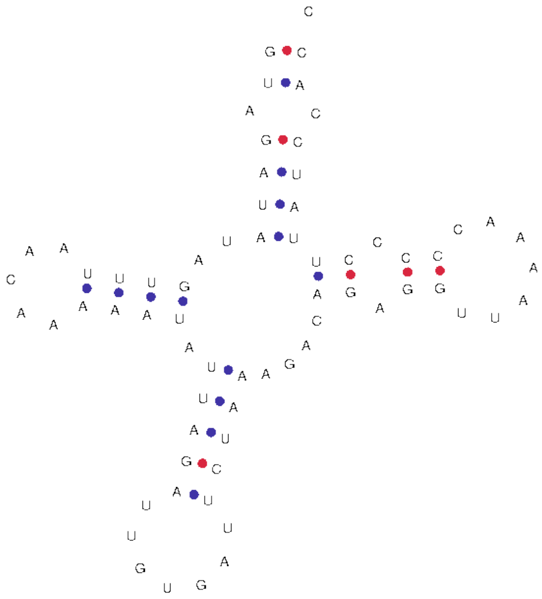

Histidine (GTG)

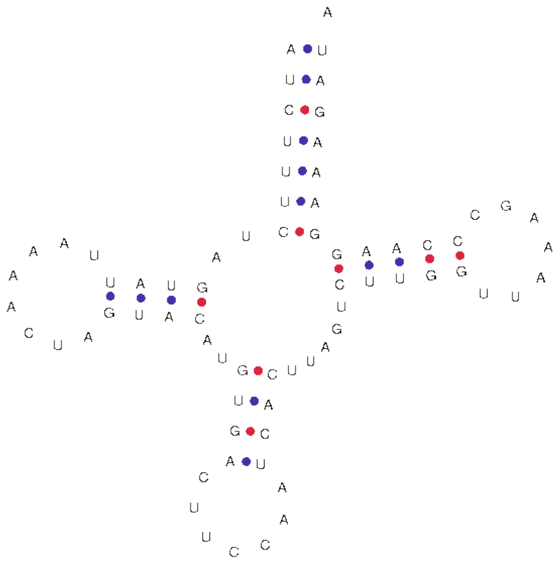

Glycine (TCC)

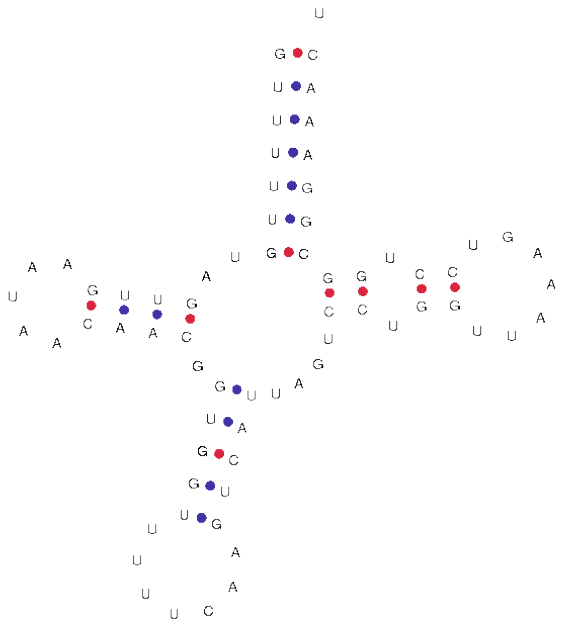

Glutamic acid (TTC)

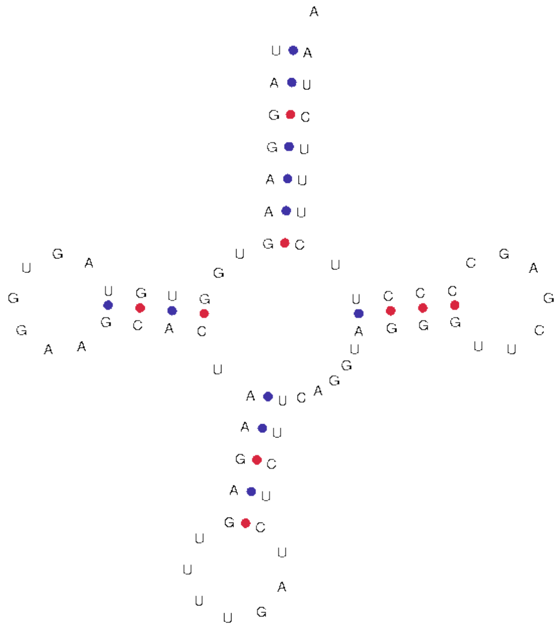

Glutamine (TTG)

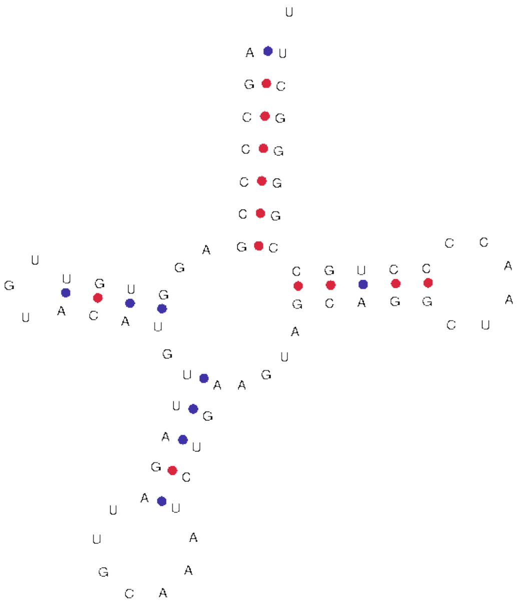

Cysteine (GCA)

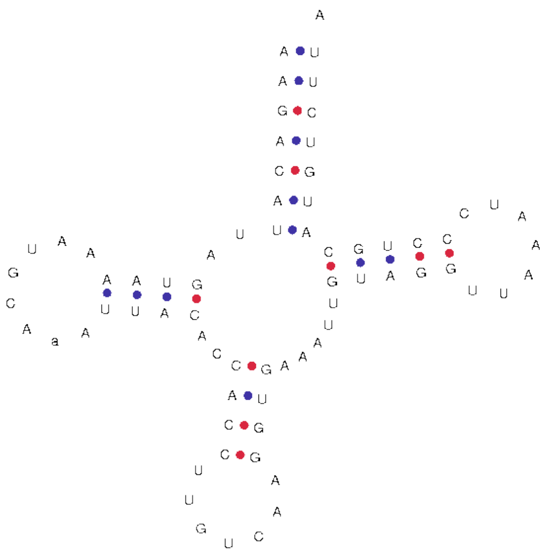

Aspartic acid (GTC)

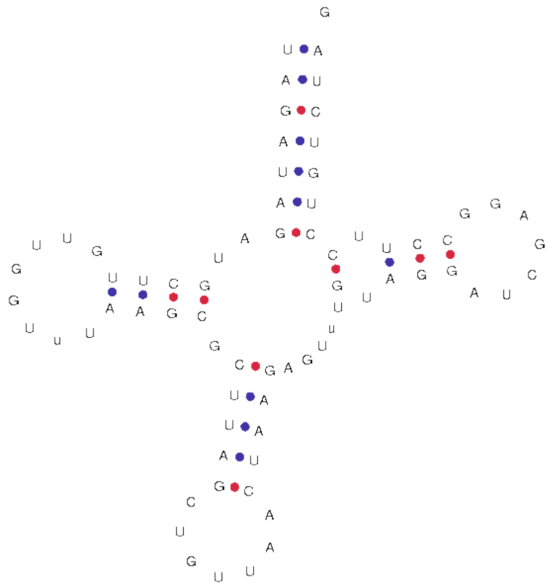

Asparagine (GTT)

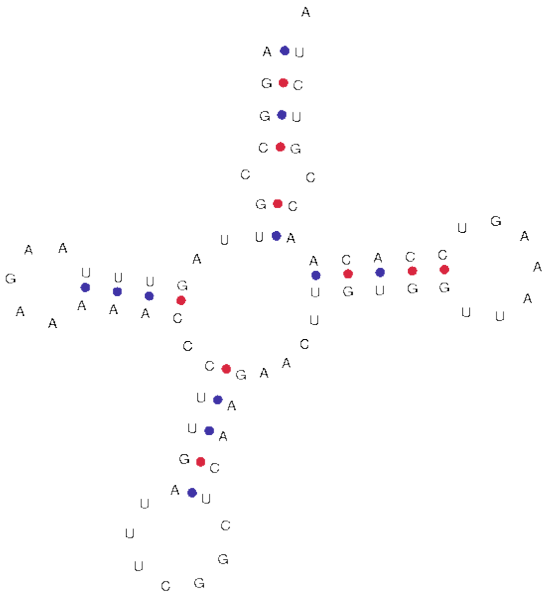

Arginine (TCG)

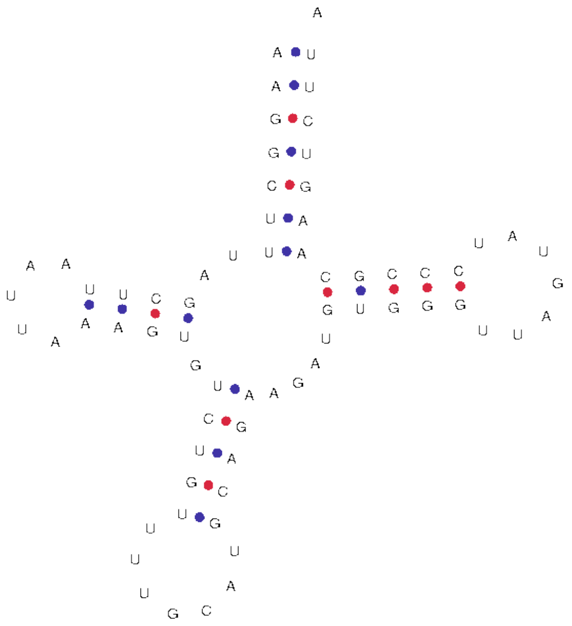

Alanine (TGC)

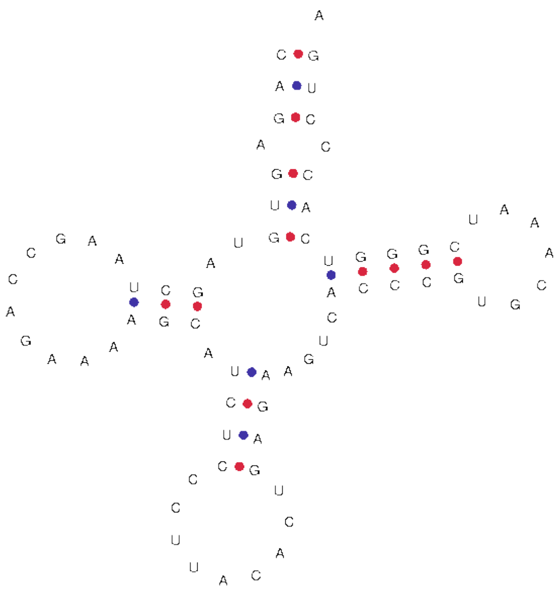

Valine (TAC)

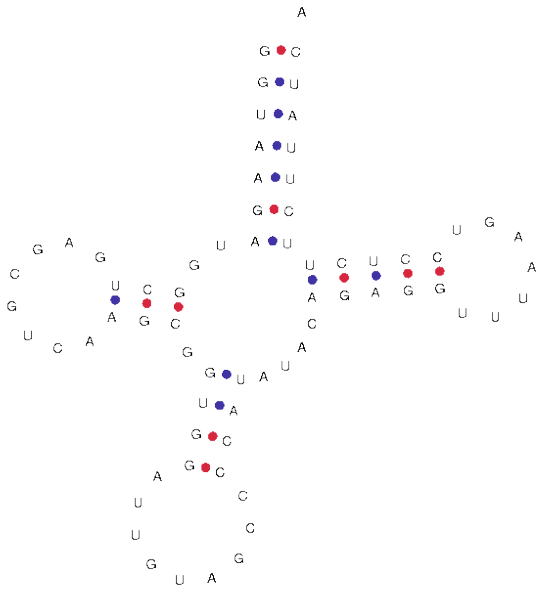

Tyrosine (GTA)

(B)

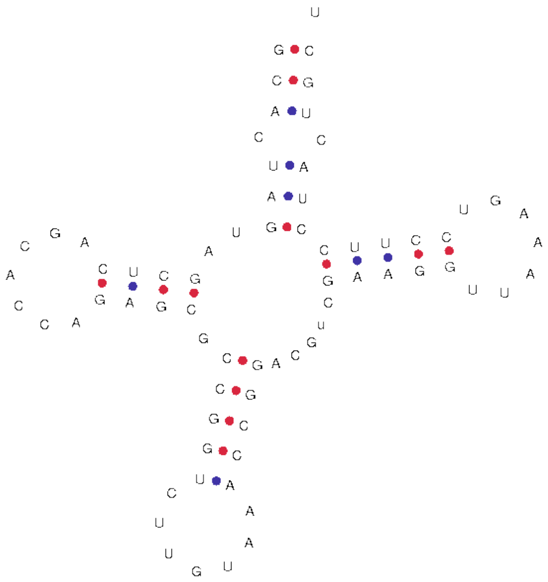

Threonine (TGT)

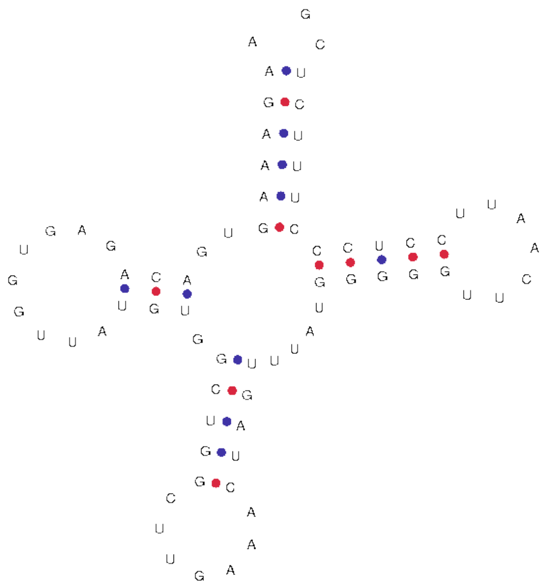

Serine (TGA)

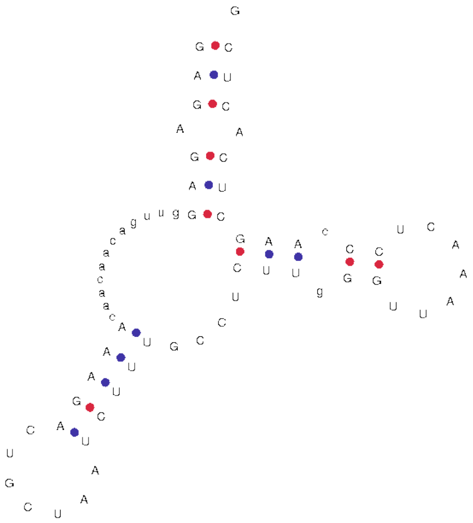

Serine (GCT)

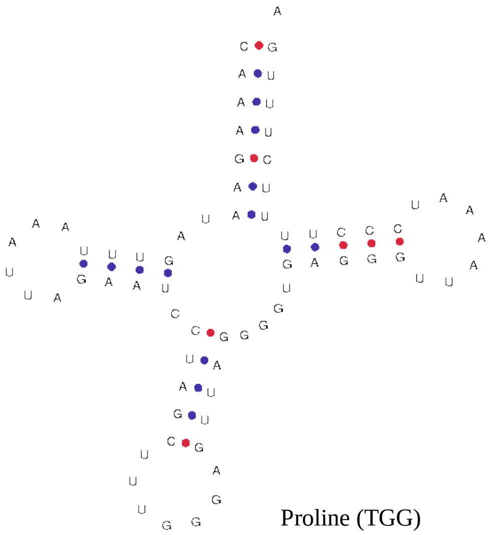

Proline (TGG)

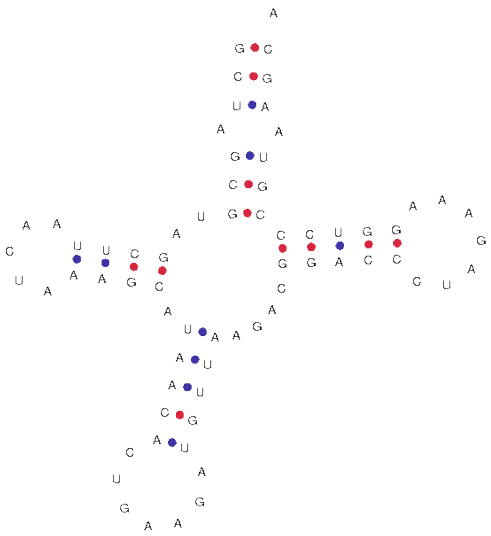

Phenylalanine (GAA)

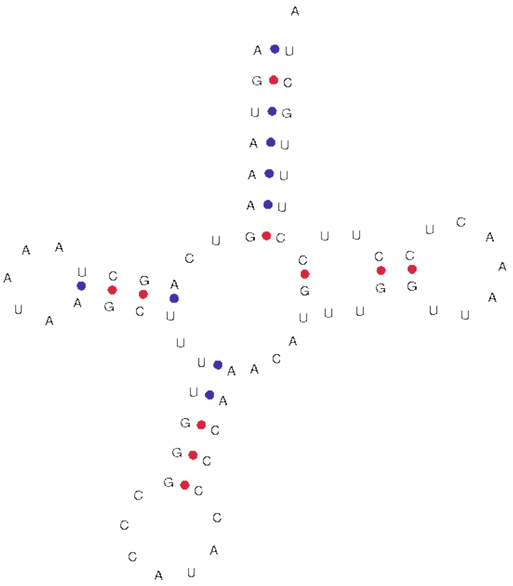

Methionine (CAT)

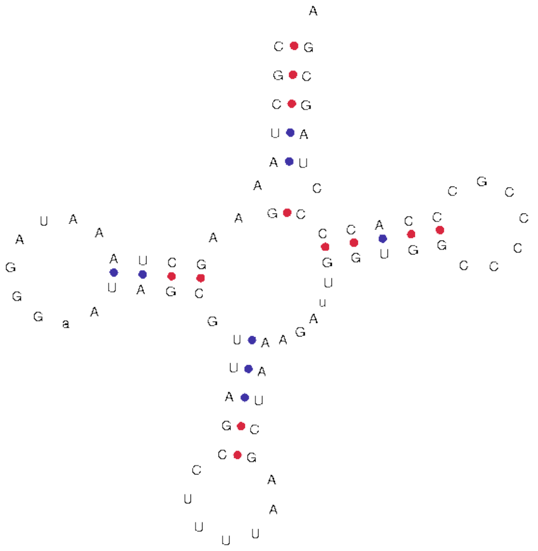

Lysine (TTT)

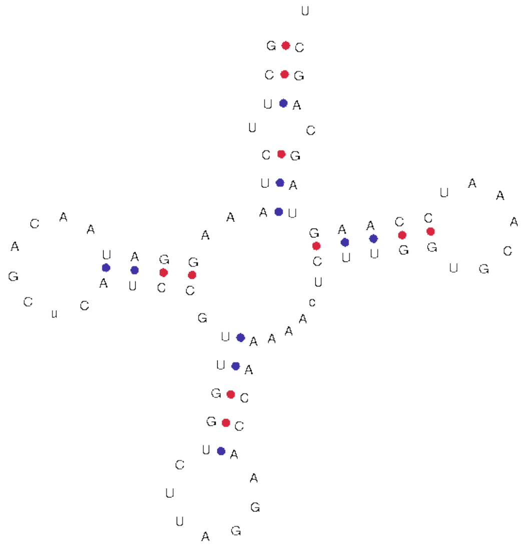

Leucine (TAG)

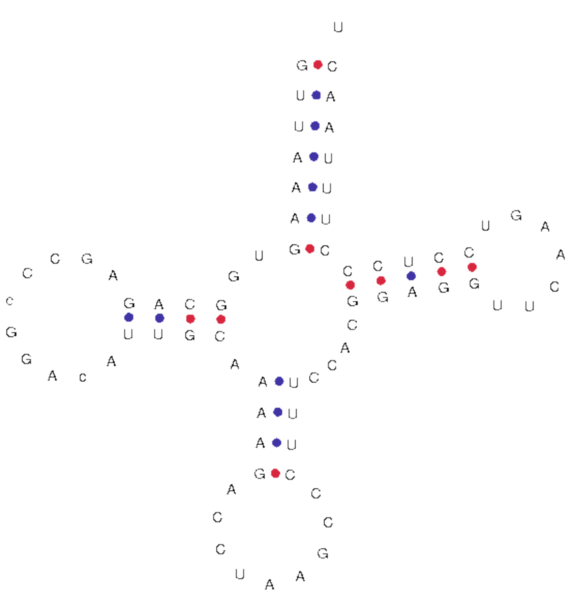

Leucine (TAA)

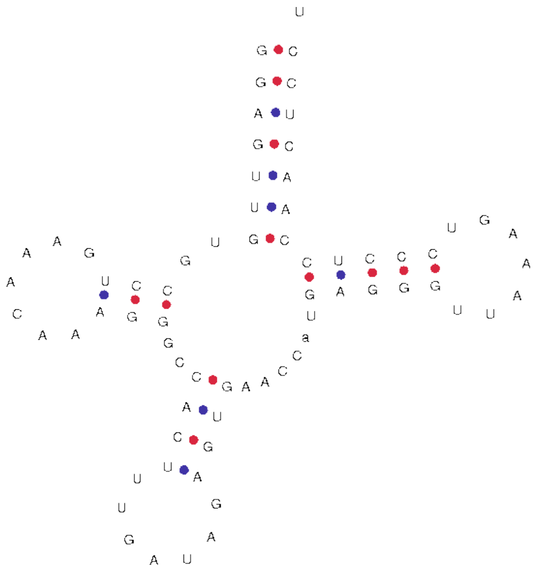

Isoleucine (GAT)

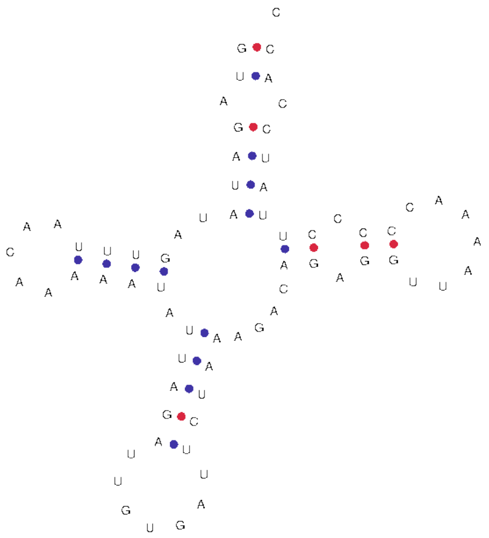

Histidine (GTG)

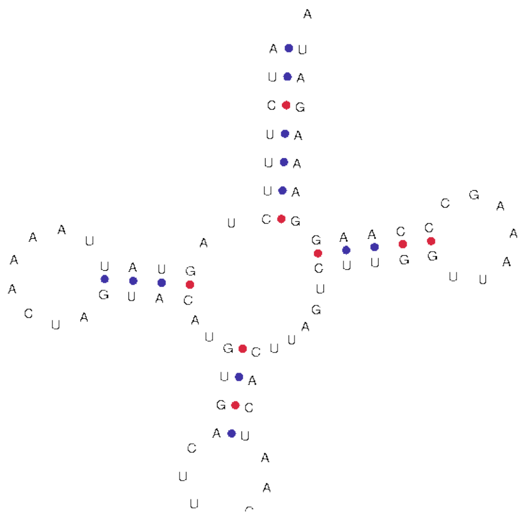

Glycine (TCC)

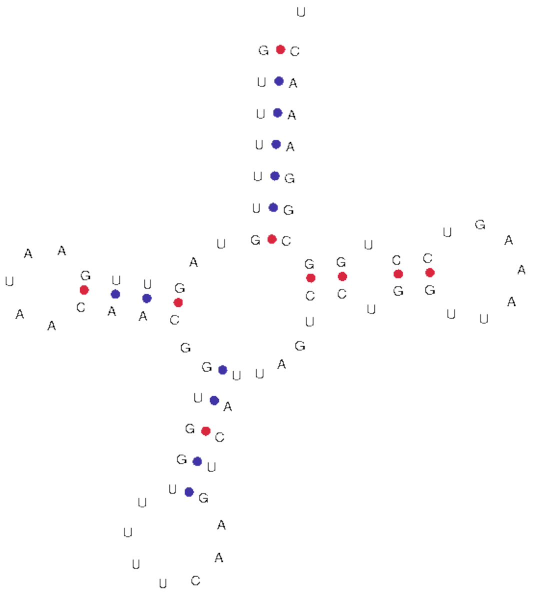

Glutamic acid (TTC)

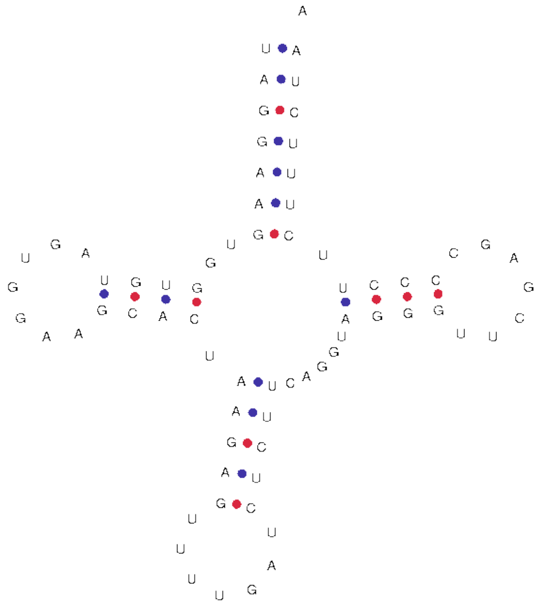

Glutamine (TTG)

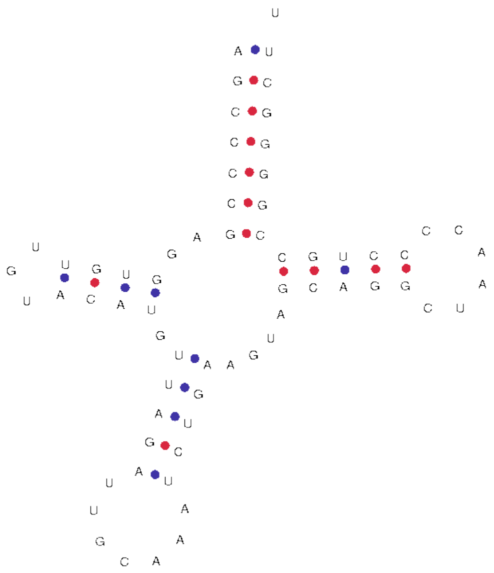

Cysteine (GCA)

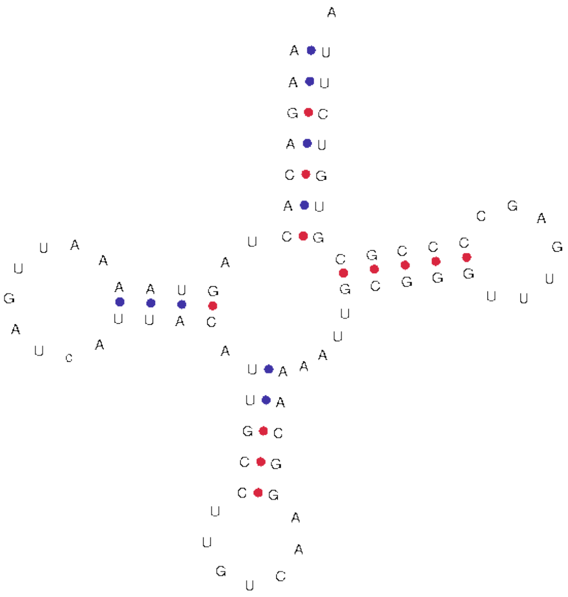

Aspartic acid (GTC)

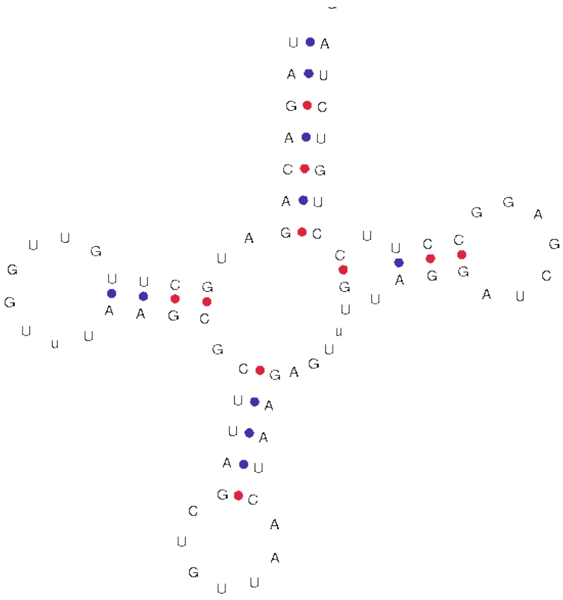

Asparagine (GTT)

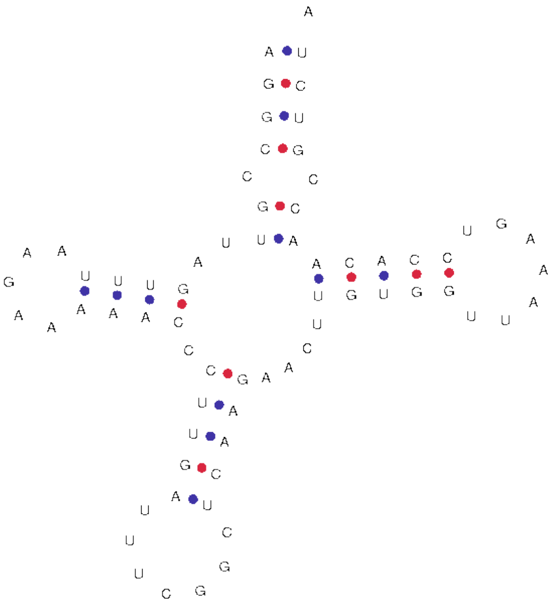

Arginine (TCG)

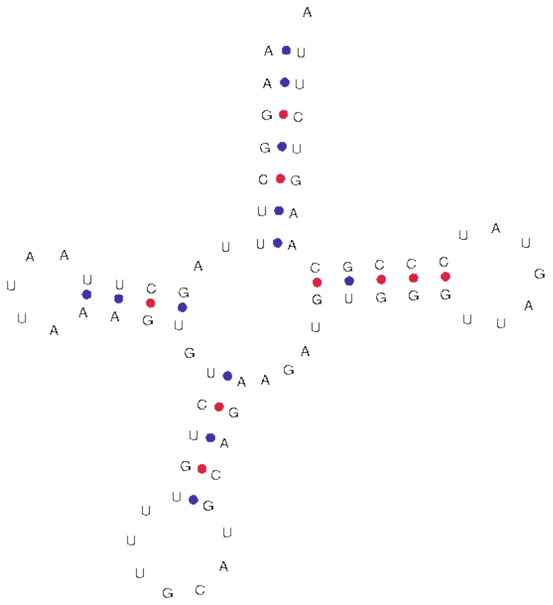

Alanine (TGC)

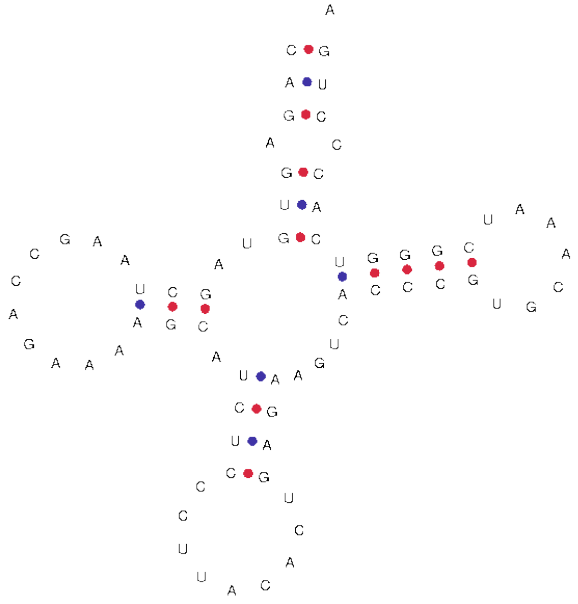

Valine (TAC)

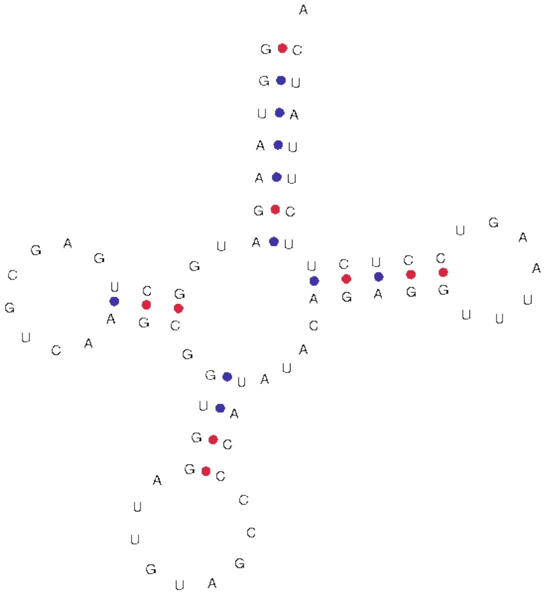

Tyrosine (GTA)

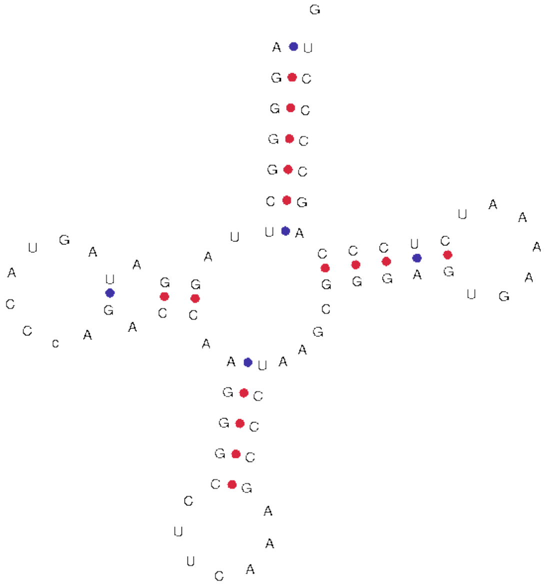

Tryptophan (TCA)

S2 Fig. Secondary structures of 22 *tRNA*s of mitochondrial genome of *O. andersonii* (A) and *O. macrochir* (B) generated by tRNAScan-SE 2.0
