# Supplementary material for: Mitochondrial genome annotation and phylogenetic placement of Oreochromis andersonii and O. macrochir among the cichlids of southern Africa
Source: PLoS One. 2018 Nov 27;13(11):e0203095. doi: 10.1371/journal.pone.0203095 (PMC6258479; doi:10.1371/journal.pone.0203095)
Supplement: S3 Fig — (PDF) [file pone.0203095.s003.pdf]

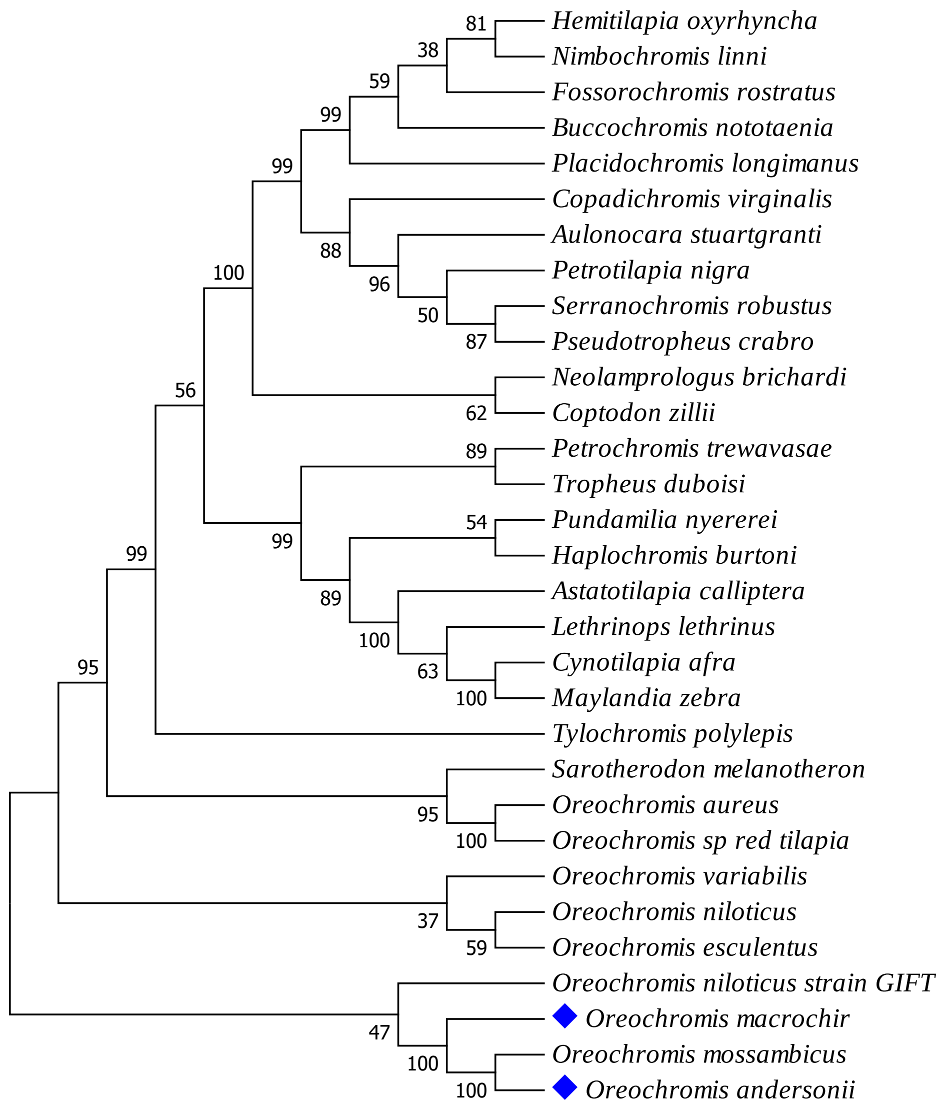

S3 Fig The phylogenetic tree showing the relative positions of *O. andersonii* and *O. macrochir* among 29 African cichlids based on Maximum Likelihood bootstrap consensus tree inferred from 1000 replicates of *ND2* gene.
