# Supplementary material for: Mitochondrial genome annotation and phylogenetic placement of Oreochromis andersonii and O. macrochir among the cichlids of southern Africa
Source: PLoS One. 2018 Nov 27;13(11):e0203095. doi: 10.1371/journal.pone.0203095 (PMC6258479; doi:10.1371/journal.pone.0203095)
Supplement: S1 Table — (PDF) [file pone.0203095.s004.pdf]

S1 Table. Designed primers used for amplification and sequencing of complete mitogenome of *O. andersonii* and *O. macrochir*

Forward	Sequence (5' to 3')	Reverse	Sequence (5' to 3')
MTON01F	CTAGAAAGGCCCCGTAAGCA	MTON01R	AGAGGGTGAGGTTTAACGGG
MTON02F	CCCGGGTACTACGAGCACTA	MTON02R	CCCTTGCGGTACTTTCTCTG
MTON03F	AACCCCAAACCCACCCTATC	MTON03R	TGGTGTGTAGTGTTCGAGCT
MTON31F	GACCACCCCGTCTCTGTG	MTON31R	TTTAGGGGTACAAGAGGCGA
MTON04F	GCCTAAAAGCAGCCACCTAC	MTON04R	GCCCCAACCAAAGACATTAG
MTON05F	CTTCAGACGCCAGAACAGAC	MTON05R	TGAGAGCATAGTTAAAGGGAGGA
MTON32F	AGAGCCCATATCGACAAGAGG	MTON32R	GGAAAGGGAAGGGGTATTGGA
MTON06F	CCCGGACATTGCAAAAGACC	MTON06R	CATATAGCGGCTAAAGGTCAGG
MTON07F	TCGCACAGACTATTTCCTACGA	MTON07R	TCTTCAGGTAGGGTTCGAGC
MTON08F	CCCCACAACTATAGCCTGGA	MTON08R	GAAGAATTAGGGCGAAGGGG
MTON09F	ATGCTTGACTCCCCGAAGTT	MTON09R	TGGGTGGAAGGTAAACGTCA
MTON10F	CGCCTCTCCTACGCAATAAC	MTON10R	CCCGAATTAGGAGGCTTAGTG
MTON11F	GACGCTCAGCCATCTTACCT	MTON11R	GGCAGGGTCAAAGAAGGTTG
MTON12F	ACCCCTATTTGTGTGATCCGT	MTON12R	GGGAATCAGTGGACGAAGCC
MTON13F	ACCGGAATTGTTCTAGCCAA	MTON13R	TGACAGAGTGGTTATGTGGCT
MTON14F	CCCCTCAAAATCCCACTAACG	MTON14R	CTAGACTTGGGACGGCTCAG
MTON15F	GGTTCCAGTTGAGTCTCCCA	MTON15R	TCAACGTGTGGTGGGTTTTG
MTON16F	TATGAGCCCCACATACCTGG	MTON16R	GCTTGATGGGCCATTAGACG
MTOM17F[a]	RCCTACAGTRGCWATYCTRACY	MTOM17R[a]	AGGAAGGTGAARTAGAAGCCT
MTON18F	ACTCAACACAGCCGTCCTAT	MTON18R	CAACGAGGAAGAAGCGAAGG
MTON19F	CCCCAAATAACCCCTGACCA	MTON19R	GCATGATAGTGGGGAGGAGA
MTON20F	CTCGCACTACTCGTCGCTA	MTON20R	CCCATCATAGTTTGTCGGCG
MTON21F	CCCTCCTTGTTGCCCTCTTA	MTON21R	CGAGGCTGGCAATGAATCAT
MTON22F	TAACTTCCTCCGCCCTCTTC	MTON22R	GATTGAGGTGAGGTGCATAGC
MTON23F	TCACTCGCCCTGCTTCTAAA	MTON23R	GGGAAGTTGCAATTCAGGCT
MTON24F	GATGATATGGCCGTGCAGAC	MTON24R	CCGATGGTTAAGCAGGAGGA
MTON25F	CACAACTTGCCTTCCTCCAC	MTON25R	GGGGTGAGGCGATGAATAAC
MTON26F	TYGGCCTTCTYACCGCACTA	MTON26R	CGGAGGGTTGRGGTAGYGGR
MTOM27F	CCCRCCTCAAAACAACCCTG	MTOM27R	TRAAGAARAARGATGCGCCR
MTOM28F[a]	CCCACATYTGTCGAGACGTR	MTOM28R[a]	KGARATTTTGTCRGCRTCTG
MTOM29FF	GCCTAAAYTCAGAYGCYGAC	MTOM29R	TGGGAGTTAGGGGYRRGGGT
MTOM30F	CCCRTYACCGGCTGRCTAGA	MTOM30R	GCGTCRATGAAAGAATGGGGR
MTOM33F	YCCCCATTCTTTCATYGACGC	MTOM33R	GCGGAGACTTGCATGTGTAA
MTON35F[m]	TCGCACTTGGAGTTCGACTA	MTON35R[m]	TGGTTAGGGYAAGGGATTGA
MTOM36F	GCAAACGACGCACTAGTTGA	MTOM36R	TGGGAGTTAGGGGTAAGGGT

[a] primers used for amplification of *O. andersonii* only; [m] primers used for amplification of *O. macrochir* only

Created by demo-version of Universal Document Converter. Full version doesn't add this stamp.
WWW.PRINT-DRIVER.COM
