# Supplementary material for: Mitochondrial genome annotation and phylogenetic placement of Oreochromis andersonii and O. macrochir among the cichlids of southern Africa
Source: PLoS One. 2018 Nov 27;13(11):e0203095. doi: 10.1371/journal.pone.0203095 (PMC6258479; doi:10.1371/journal.pone.0203095)
Supplement: S2 Table — (PDF) [file pone.0203095.s005.pdf]

S2 Table. Complete automated annotation of *O. andersonii* and *O. macrochir* mitochondrial genome.

Gene		DOGMA				MITOS				MITOANNOTATOR		
		Position (pb)		Length (bp)[a]		Position (bp)		Length (bp)		Position (bp)		Length (bp)[a]
		Initial	Final [a]			Initial [a]	Final [a]			Initial	Final [a]	
tRNA^{Phe}		1	69	69		1	69	69		1	69	69
12S rRNA		70	**1010**	941		70	1013	944		70	1013	944
tRNA^{Val}		1014	1085	72		1014	1085	72		1014	1085	72
16S rRNA		1086	**2770**	1685		1087	2779	1695		1086	2779	1694
tRNA^{Leu}		2780	2853	74		2780	2853	74		2780	2853	74
**ND1**		**2854**	**3825**	**972**		**2869**	**3822**	**954**		**2854**	**3828**	**975**
tRNA^{Ile}		3832	3901	70		3832	3901	70		3832	3901	70
tRNA^{Gln}		3901	3971	71		3901	3971	71		3901	3971	71
tRNA^{Met}		3971	4039	69		3971	4039	69		3971	4039	69
**ND2**		**4040**	**5092/5089**	**1053/1050**		**4040**	**5086**	**1047**		**4040**	**5094**	**1055**
tRNA^{Trp}		5095	5166	72		5095	5166	72		5095	5166	72
tRNA^{Ala}		5168	5236	69		5168	5236	69		5168	5236	69
tRNA^{Asn}		5238	5310	73		5238	5310	73		5238	5310	73
tRNA^{Cys}		5344	5409	66		5344	5409	66		5344	5409	66
tRNA^{Tyr}		5410	5479	70		5410	5479	70		5410	5479	70
**COI**		**5481**	**7034**	**1554**		**5487**	**7028**	**1542**		**5481**	**7082**	**1602**
tRNA^{Ser}		7083	7153	71		7083	7153	71		7083	7153	71
tRNA^{Asp}		7157	7229	73		7157	7229	73		7157	7229	73
**COII**		**7235**	**7924**	**690**		**7235**	**7918**	**684**		**7235**	**7925**	**691**
tRNA^{Lys}		7926	7999	74		7926	7999	74		7926	7999	74
**ATPase 8**		**8001**	**8165**	**165**		**8001**	**8165**	**165**		**8001**	**8168**	**168**
**ATPase 6**		**8159**	**8830**	**672**		**8159**	**8830**	**672**		**8159**	**8832**	**674**
**COIII**		**8833**	**9615**	**783**		**8833**	**9615**	**783**		**8833**	**9616**	**784**
tRNA^{Gly}		9617	9688	72		9617	9688	72		9617	9688	72
**ND3**		**9689**	**10036**	**348**		**9689**	**10036**	**348**		**9689**	**10037**	**349**
tRNA^{Arg}		10038	10106	69		10038	10106	69		10038	10106	69
**ND4L**		**10107**	**10400**	**294**		**10107**	**10400**	**294**		**10107**	**10403**	**297**
**ND4**		**10397**	**11785**	**1389**		**10397**	**11776**	**1380**		**10397**	**11786**	**1390**

Created by demo-version of Universal Document Converter. Full version doesn't add this stamp.
WWW.PRINT-DRIVER.COM

tRNA^{His}		11787	11855	69		11787	11855	69		11787	11855	69
tRNA^{Ser}		11856	11922	67		11856	11922	67		11856	11922	67
tRNA^{Leu}		11927	11999	73		11927	11999	73		11927	11999	73
**ND5**		**12000**	**13808**	**1809**		**12018/12015**	**13805**	**1788/1791**		**12000**	**14300**	**2301**
**ND6**		**13843**	**14361**	**519**		**13843**	**14361**	**519**		**13840**	**14361**	**522**
tRNA^{Glu}		14362	14430	69		14362	14430	69		14362	14430	69
**Cyt b**		**14435**	**15571**	**1137**		**14435**	**15568**	**1134**		**14435**	**15575**	**1141**
tRNA^{Thr}		15576	15647	72		15576	15647	72		15576	15647	72
tRNA^{Pro}		15648	15717	70		15648	15717	70		15648	15717	70
CR		15718	16642/16644	925/927		15718	16642/16644	925/927		15718	16642/16644	925/927

[a] The forward slashes (/) denote the values of *O. andersonii*/ *O. macrochir*, if none the values for both species are identical.

Created by demo-version of Universal Document Converter. Full version doesn't add this stamp.
WWW.PRINT-DRIVER.COM
