# Supplementary material for: Mitochondrial genome annotation and phylogenetic placement of Oreochromis andersonii and O. macrochir among the cichlids of southern Africa
Source: PLoS One. 2018 Nov 27;13(11):e0203095. doi: 10.1371/journal.pone.0203095 (PMC6258479; doi:10.1371/journal.pone.0203095)
Supplement: S3 Table — (PDF) [file pone.0203095.s006.pdf]

S3 Table. Codon usage of complete mitogenome sequences of *Oreochromis andersonii* (16642 bp) and *O. macrochir* (16644 bp).

		Count		RSCU				Count		RSCU	
Amino acid	Codon	*O.A	*O.M	O.A	O.M	Amino acid	Codon	O.A	O.M	O.A	O.M
Phenylalanine (Phe/F)	UUU	127	126	1.04	1.04	Tyrosine (Try/Y)	UAU	95	98	0.91	0.95
	UUC	118	116	0.96	0.96		UAC	113	108	1.09	1.05
Leucine (Leu/L)	UUA	122	126	1.12	1.15	Stop	UAA(*)	115	118	1.28	1.33
	UUG	82	79	0.75	0.72		UAG(*)	79	77	0.88	0.87
	CUU	151	152	1.38	1.39	Histidine (His/H)	CAU	118	121	0.98	0.98
	CUC	126	128	1.15	1.17		CAC	124	125	1.02	1.02
	CUA	104	101	0.95	0.92	Glutamine (Gln/Q)	CAA	148	143	1.39	1.36
	CUG	71	70	0.65	0.64		CAG	65	68	0.61	0.64
Isoleucine (Ile/I)	AUU	90	94	1.13	1.15	Asparagine (Asn/N)	AAU	132	135	0.9	0.92
	AUC	70	69	0.88	0.85		AAC	161	158	1.1	1.08
Methionine (Met/M)	AUA	78	77	1.18	1.18	Lysine (Lys/K)	AAA	152	152	1.27	1.27
	AUG	54	53	0.82	0.82		AAG	88	88	0.73	0.73
Valine (Val/V)	GUU	53	54	1.35	1.43	Aspartic acid (Asp/D)	GAU	45	46	0.89	0.9
	GUC	39	36	0.99	0.95		GAC	56	56	1.11	1.1
	GUA	37	35	0.94	0.93	Glutamic acid (Glu/E)	GAA	86	86	1.27	1.27
	GUG	28	26	0.71	0.69		GAG	49	49	0.73	0.73
Serine (Ser/S)	UCU	151	140	1.54	1.44	Cysteine (Cys/C)	UGU	39	37	0.72	0.71
	UCC	124	127	1.26	1.31		UGC	69	67	1.28	1.29
	UCA	102	103	1.04	1.06	Tryptophan (Trp/W)	UGA	71	72	1.21	1.22
	UCG	55	55	0.56	0.57		UGG	46	46	0.79	0.78
Proline (Pro/P)	CCU	226	229	1.43	1.44	Arginine (Arg/R)	CGU	38	36	0.78	0.73
	CCC	201	202	1.27	1.27		CGC	74	78	1.51	1.58
	CCA	130	127	0.82	0.8		CGA	53	51	1.08	1.04
	CCG	74	76	0.47	0.48		CGG	31	32	0.63	0.65
Threonine (Thr/T)	ACU	107	111	1.09	1.1	Serine (Ser/S)	AGU	58	59	0.59	0.61
	ACC	114	119	1.17	1.18		AGC	99	98	1.01	1.01
	ACA	128	130	1.31	1.29	Stop	AGA(*)	85	82	0.95	0.92
	ACG	42	44	0.43	0.44		AGG(*)	79	79	0.88	0.89
Alanine (Ala/A)	GCU	49	50	0.98	0.98	Glycine (Gly/G)	GGU	39	37	0.89	0.85
	GCC	87	85	1.74	1.67		GGC	49	50	1.12	1.15
	GCA	44	47	0.88	0.92		GGA	44	45	1.01	1.03
	GCG	20	22	0.4	0.43		GGG	43	42	0.98	0.97

RSCU = Relative synonymous codon usage

O.A= Oreochromis andersonii   *O.M= Oreochromis macrochir*
